# Route of *Francisella tularensis* infection informs spatiotemporal metabolic reprogramming and inflammation in mice

**DOI:** 10.1371/journal.pone.0293450

**Published:** 2023-10-26

**Authors:** Forrest Jessop, Benjamin Schwarz, Eric Bohrnsen, Catharine M. Bosio

**Affiliations:** Rocky Mountain Laboratories, NIAID, Hamilton, MT, United States of America; Federal University Dutse, NIGERIA

## Abstract

Route of exposure to pathogens can inform divergent disease pathogenesis and mortality rates. However, the features that contribute to these differences are not well established. Host metabolism has emerged as a critical element governing susceptibility and the metabolism of tissue exposure sites are unique. Therefore, specific metabolic niches may contribute to the course and outcome of infection depending on route of infection. In the current study, we utilized a combination of imaging and systems metabolomics to map the spatiotemporal dynamics of the host response to intranasal (i.n.) or intradermal (i.d.) infection of mice using the bacterium *Francisella tularensis subsp tularensis* (FTT). FTT causes lethal disease through these infection routes with similar inoculation doses and replication kinetics, which allowed for isolation of host outcomes independent of bacterial burden. We observed metabolic modifications that were both route dependent and independent. Specifically, i.d. infection resulted in early metabolic reprogramming at the site of infection and draining lymph nodes, whereas the lungs and associated draining lymph nodes were refractory to metabolic reprogramming following i.n. infection. Irrespective of exposure route, FTT promoted metabolic changes in systemic organs prior to colonization, and caused massive dysregulation of host metabolism in these tissues prior to onset of morbidity. Preconditioning infection sites towards a more glycolytic and pro-inflammatory state prior to infection exacerbated FTT replication within the lungs but not intradermal tissue. This enhancement of replication in the lungs was associated with the ability of FTT to limit redox imbalance and alter the pentose phosphate pathway. Together, these studies identify central metabolic features of the lung and dermal compartments that contribute to disease progression and identify potential tissue specific targets that may be exploited for novel therapeutic approaches.

## Introduction

The metabolic milieu of a tissue can influence the timing and/or composition of the immune response needed to counter invading pathogens. However, we currently do not understand if the relative propensities of different tissues to metabolically adapt and promote immune activation influences disease pathogenesis to morbidity. It is possible that differences in primary exposure site and/or the associated localized metabolic response (including timing, composition, and magnitude) could contribute to divergent outcomes with respect to disease severity. Such deviations in adaptation could have significant impacts on how we design or implement lifesaving therapeutic interventions.

The highly virulent intracellular bacterium *Francisella tularensis* subsp *tularensis* (FTT) is known to infect via several routes including the lungs, gastrointestinal tract, eye, and intradermal compartment [1]. Dissemination from these tissues and establishment of sepsis is associated with poor outcomes [2]. However, in humans pulmonary infection is specifically associated with more severe disease and higher mortality rates compared to dermal infections (30–60% versus 5%, respectively) [3,4]. The stark differences between outcomes of pulmonary versus intradermal infection implicates evasion and/or specific manipulation of metabolic and immune defenses in the lung that may otherwise be activated within the intradermal compartment resulting in greater opportunity for resolution of infection. For example, increased and/or earlier inflammatory responses in the intradermal tissue could lead to earlier symptom manifestation and acquisition of life-saving antibiotic therapy whereas this host response has been shown to be largely absent in the lung. Specific metabolic host factors contributing to disparate outcomes after FTT infection between pulmonary and intradermal infection have not been fully elucidated. Identification of these elements and their associated host pathways will allow for improved delivery of existing countermeasures and/or inform development of novel therapeutics or vaccine strategies.

As indicated above, pulmonary infection with FTT is underscored by the ability of the bacterium to evade and suppress pro-inflammatory cascades from early to mid-phase of infection creating an environment conducive to unchecked replication [5,6]. We have demonstrated that FTT specifically manipulates aspects of central metabolism in vitro and in vivo that would normally be engaged to support pro-inflammatory activity as an integral facet of its ability to evade and suppress this host response [7–10]. This includes limiting stabilization of HIF-1α and reprogramming of components within the glycolytic pathway known to support inflammatory cytokine production [11]. FTT also supports increased mitochondrial respiration to limit early host cell death, allowing for greater replication [12]. However, the full extent of manipulation of central metabolism by FTT at the whole tissue level is not understood, including whether the capacity of FTT to manipulate metabolism may differ when infection is initiated in the lungs versus a peripheral location like the dermis due to different basal metabolic milieus in those compartments. Additionally, how the metabolic response progresses in systemic organs immediately prior and after colonization has not been characterized.

In the current study, we compared the spatiotemporal metabolic response to pulmonary versus intradermal FTT infection. We specifically exploited the conserved feature that infection with 25 CFU causes lethality in mice initiated through the pulmonary or intradermal routes, thereby controlling for the contribution of dose to magnitude or timing of the host response. We hypothesized that the propensity of the lungs to undergo metabolic reprogramming would be reduced and/or undergo greater manipulation by FTT compared to the intradermal compartment after infection leading to a delayed or reduced inflammatory response at the site of infection. Consistent with this hypothesis, comparative analyses revealed key differences in the timing of metabolic reprogramming between the lungs and intradermal compartment at early time points of infection and prior to escape of bacteria into the periphery. However, after dissemination to peripheral tissues, the metabolic response was highly conserved between infection routes across multiple organs. To identify metabolic networks actively manipulated by FTT that could account for increased susceptibility of the lungs to early infection and worse prognosis we systemically preconditioned the host with LPS to provoke pro-inflammatory metabolic phenotype prior to infection. Remarkably, LPS preconditioning enhanced FTT infection in the lung, but not intradermal compartment. Enhanced susceptibility of the lungs was strongly associated with active manipulation of glycolytic intermediates, the pentose phosphate pathway, as well as redox and xanthine metabolism. Together, these data show that FTT overcomes complex metabolic barriers in multiple organs to promote a highly conserved metabolic phenotype. Further, they also reveal that FTT differentially modifies components of central metabolism in the lung after pulmonary exposure to achieve greater burdens early in infection relative to the intradermal inoculation site.

## Materials and methods

### Bacterial Strains

FTT SchuS4 was obtained from Dr. Jeannine Peterson, CDC, Ft. Collins and generated into single use stocks as previously described [13]. All experiments were performed under approved biosafety level 3 protocols at Rocky Mountain Laboratories.

### Mice

All studies were performed using female C57Bl/6 mice between 6–9 weeks old. Mice were purchased from Jackson Laboratories and housed in animal biosafety level 3 facilities at Rocky Mountain Laboratories. Mice were housed in sterile cages and provided irradiated food and sterile water *ad libitum*. Housing was in a specific-pathogen-free facility with 12 hr light/dark cycles. All animal studies were approved by the Rocky Mountain Laboratories Animal Care and Use Committee and performed in compliance with their guidelines (ASP # 2019-054-E).

### In vivo studies

Mice were anesthetized using ketamine/xylazine prior to infection. Intranasal and intradermal infections were performed as previously described [14]. Briefly, FTT stocks were diluted in PBS to either 25 CFU/25μL (intranasal) or 25 CFU/10 μl (intradermal). For intranasal infections, inoculum was instilled down the left nare of the anesthetized mouse. For intradermal infection, inoculum was loaded into a tuberculin syringe and approximately 10 μL was injected into the ear pinna of the anesthetized mouse. Mice injected with PBS served as negative (mock) controls. Concentration of inoculums was confirmed by plating on Modified Mueller Hinton (MMH) agar plates followed by incubation and enumeration of colonies. LPS preconditioning of mice was accomplished by intraperitoneal injection (i.p.) of a sublethal bolus of TLRGRADE® LPS from E. coli Serotype O111:B4 (EnzoLife Sciences) at 500 mg/kg. After 24 hours, mice were infected with FTT as described above. At 24 hours post infection, mice were euthanized, and tissues processed for enumeration of bacterial burden, inflammatory cytokine levels, and metabolites.

### In vivo imaging

Mice were anesthetized and thorax and belly shaved 2 days prior to infection with FTT. Mice were housed on diamond bedding (ENVIGO) to limit dermal abrasions that can cause superficial inflammation and interfere with deep tissue signals. Two hours prior to imaging, mice were injected via the retro-orbital route with 100 μL XenoLight RediJect 2-DeoxyGlucosone 750 (RJ2-DG-750, PerkinElmer) or AnnexinVivo 750 (PerkinElmer). Near-infrared imaging of fluorescent probe distribution and intensity was carried out using an IVIS Lumina XR instrument (PerkinElmer). Immediately prior to imaging, the shave regions on the mice were delipidated using Nair© (Nair, Church and Dwight, NJ) to remove any residual hair that could interfere with the deep tissue fluorescent signal. To image, mice were anesthetized using 1.5% isoflurane/oxygen air mixture and positioned on the imaging platform within the IVIS Lumina XR in the supine position. Black masking tape (Thor Labs) and black construction paper were used to mask signal from regions of non-interest including the bladder, hands, feet, nose, and tail. Images were acquired with indocyanine green filter sets with Ex 745nm/Em820 nm. Immediately following acquisition of whole-body images, mice were euthanized, and individual tissues excised and imaged ex vivo in a petri dish to resolve anatomical specificity of signal. Image analysis was done using Living Image 4.7 software (PerkinElmer).

### Enumeration of bacterial burden

Lungs, liver, spleens, lymph nodes, and ears were homogenized following methods previously described [15]. Briefly, tissues were homogenized in ice cold tissue lysis buffer (150 mM Tris-HCL, 5 mM EDTA, 10 mM Trizma base) containing protease inhibitor cocktails I, II, and III (Ag Scientific) by grinding through a fine wire mesh (Belleville Wire Cloth Co.). Tissue homogenates were then serially diluted and plated onto MMH agar plates. MMH agar plates were incubated at 37°C with 5% CO_2_ and colonies enumerated after 48 hours.

### Quantification of host cytokines

Tissue homogenates were centrifuged at 10,000 x g for 20 minutes to clarify allowing for isolation of the soluble fraction. IFN-γ, IL-6, MCP-1, MIP-1α, TNF-α, KC, RANTES, IL-12p70, EPO, IFN-β, or MMP-9 were measured in the whole tissue homogenates using a Cytometric Bead Array (CBA; BD Biosciences) or Biomarker Group 1 Mesoplex Array (MesoScale) according to manufactures instructions. For the CBA, beads were fixed in PFA prior to flow cytometric analysis. IL-12p40 was measured by ELISA (BD Biosciences) according to manufactures instructions.

### LC-MS

For all liquid chromatography–mass spectrometry (LC-MS) analyses, LC-MS–grade solvents and reagents were used. The post caval lobe of the lung, the median lobe of the liver, half the spleen, the ear, and mediastinal or cervical lymph nodes of mice were collected into 400 μL of ice-cold methanol. Tissues were subsequently bead homogenized, and 400 μL of water added. To the suspension 400 μL of chloroform was added, and the sample was agitated via shaking at 4°C for 20 minutes. Layering was induced by centrifugation at 16,000 x *g*, 4°C for 20 minutes. The polar (upper) layer was collected and diluted 1:10 in 50% methanol in water. Analysis was performed consistent with previous reports [16]. Samples were injected onto a Sciex ExionLC AC system and separated using an ion pairing strategy on a Waters Atlantis T3 column (100 Å, 3 μm, 3 mm × 100 mm) with a 15-minute gradient from 5 mM tributylamine, 5 mM acetic acid in 2% isopropanol, 5% methanol, 93% water (vol/vol) to 100% isopropanol. Metabolites were detected on a Sciex 5500 QTRAP mass spectrometer using a MRM strategy consistent with previous reports [16]. QC injections were performed after every 10 injections to ensure instrument stability. All data were processed and filtered using MultiQuant Software 3.0.3 (Sciex). A 50% missing value cutoff and 30% QC coefficient of variance cutoff were imposed. Remaining missing values were replaced with the minimum group value for that feature. Following filtering, the data was normalized to either the corresponding day 0 or mock-infected vehicle control groups and scaled as the log_2_ of the fold change versus the mean of the reference group. A Benjamini-Hochberg correction was applied to univariate analysis with a 10% FDR cutoff. All univariate and multivariate analysis was performed in MarkerView Software 1.3.1 (Sciex).

### Statistical analysis

**Fig 1:**
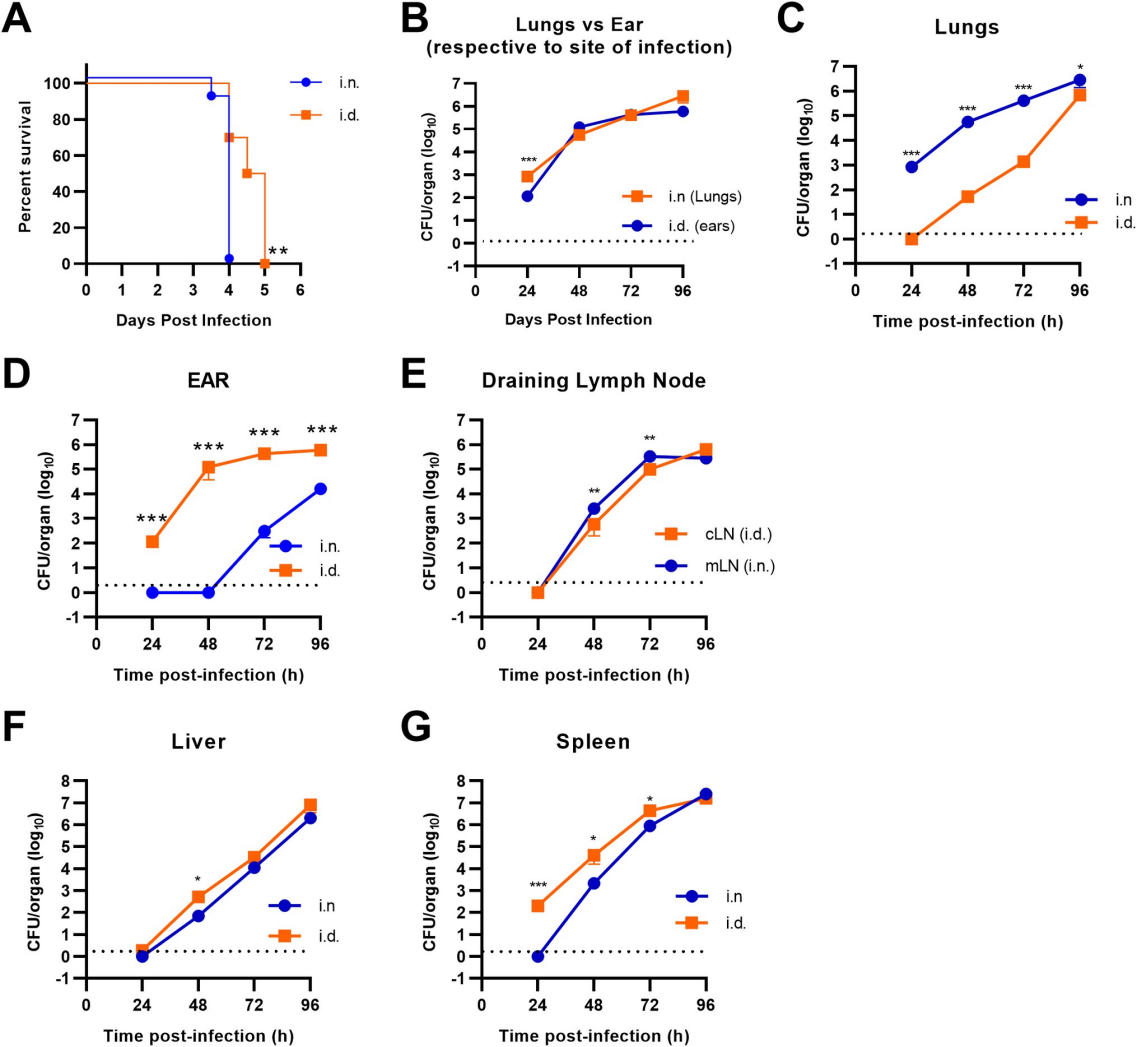
Bacterial replication and dissemination following pulmonary or intradermal infection. (A) Mice were infected with FTT via i.d. or i.n. route with FTT (25 CFU) and survival assessed over time. (B) FTT burden in the lungs after i.n. infection or ears after i.d. infection. (C) FTT burden in the lungs of i.n or i.d. infected mice. (D) FTT burden in the ear of i.n. or i.d. infected mice. (E). Bacterial burden in the cLN after i.d. infection or mLN after i.d. infection. Bacterial burdens in the liver (F) and spleen (G). Dotted black line was the limit of detection. Data are shown as the combined results from two separate experiments (N = 9–10). Data are shown as mean +/- SEM. A Log-rank (Mantel-Cox) test was used to determine significance in survival between i.n. and i.d. infected mice. *p<0.05, **p<0.01, ***p<0.001 indicates significance between i.n. and i.d. infection using an unpaired t-test corrected for multiple comparisons using the Holm-Sidak method.

**Fig 2:**
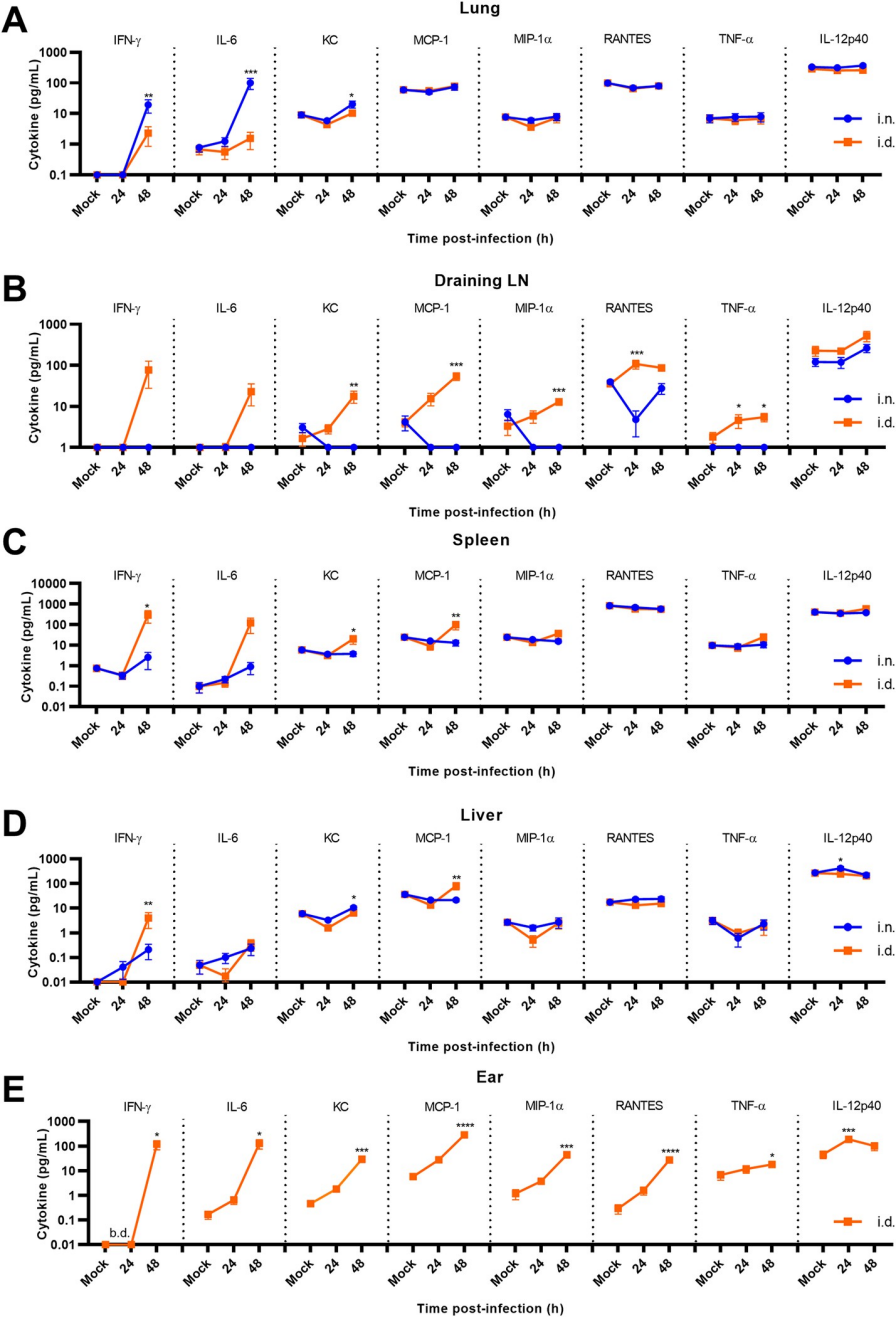
Comparative analysis of inflammatory cytokine/chemokine response following intranasal or intradermal infection. Whole tissue homogenates were evaluated for changes in cytokine/chemokine levels in the (A) lungs, (B) mLN or cLN, (C) spleen, (D) liver, and (E) ear (i.d. infection only). Data shown are mean ± SEM and were pooled from two separate experiments (N = 10 mice per group). *p<0.05, **p<0.01, ***p<0.001 indicates significance between i.n. and i.d. infection using an unpaired t-test corrected for multiple comparisons using the Holm-Sidak method. For the ear after i.d. infection (B), *p<0.05, **p<0.01, ***p<0.001 indicates significance to time mock infected controls using a one-way ANOVA followed by Dunnett’s correction for multiple comparisons.

**Fig 3:**
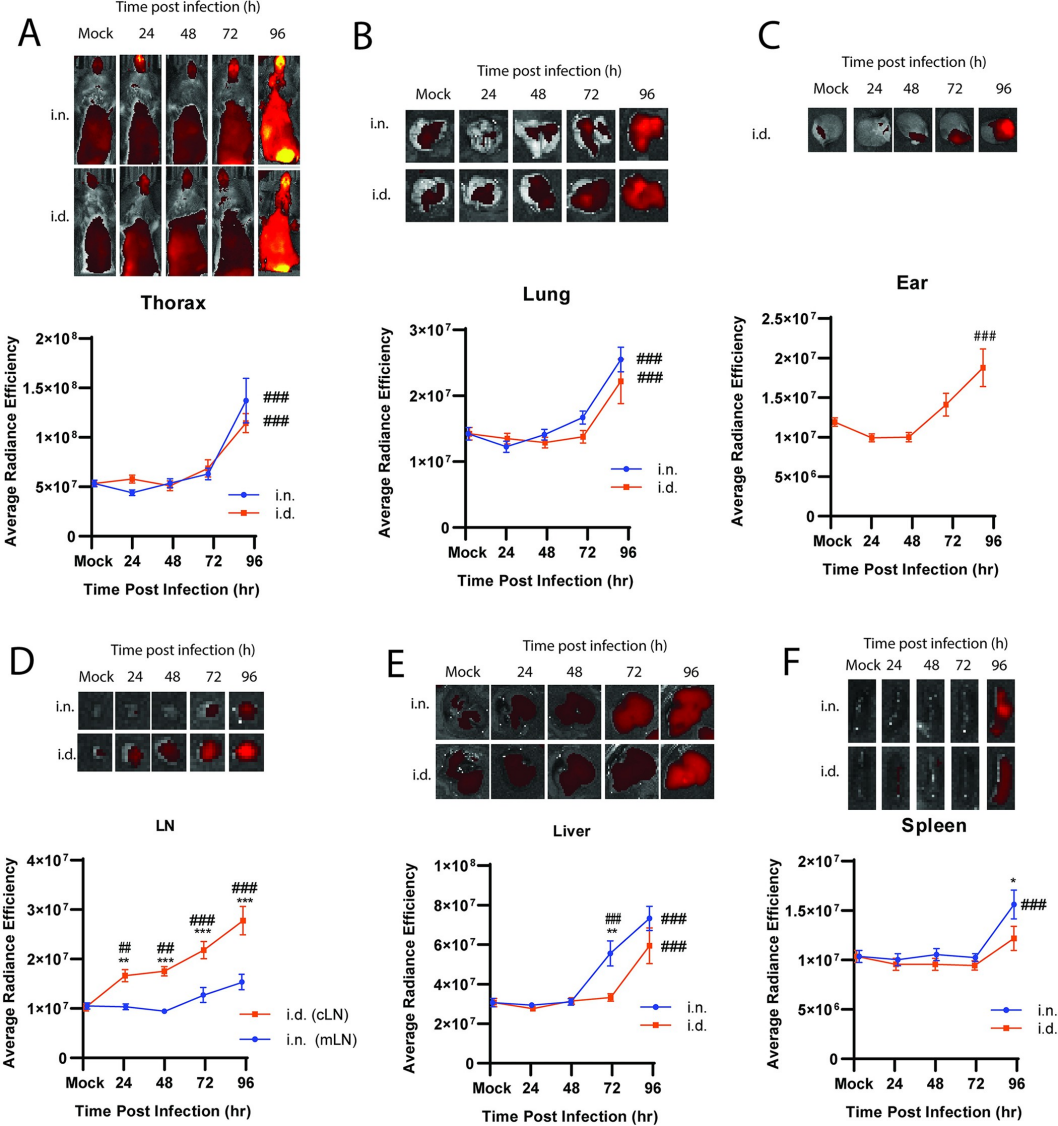
Glucose uptake and distribution following FTT infection. Mice infected i.d. or i.n. with FTT were injected i.v. with RJ2-DG-750 and distribution quantified at the whole animal level (A). Tissues were removed and imaged ex vivo for tissue specific resolution of the (B) lungs, (C) ear (after i.d. infection only), (D) mLN or cLN, (E) liver, and (F) spleen. Data shown are mean +/- SEM of the average radiance efficiency and an accompanying representative image from two separate experiment (N = 10 mice per group). *p<0.05, **p<0.01, ***p<0.001 indicate significance between i.d. and i.n. using an unpaired t-test corrected for multiple comparisons using the Holm-Sidak method (A-B, D-F). #p<0.05, ##p<0.01, ###p<0.001 indicate significance relative to mock infected control using a one or 2-way ANOVA corrected for multiple comparisons using the Dunnett’s post-test.

**Fig 4:**
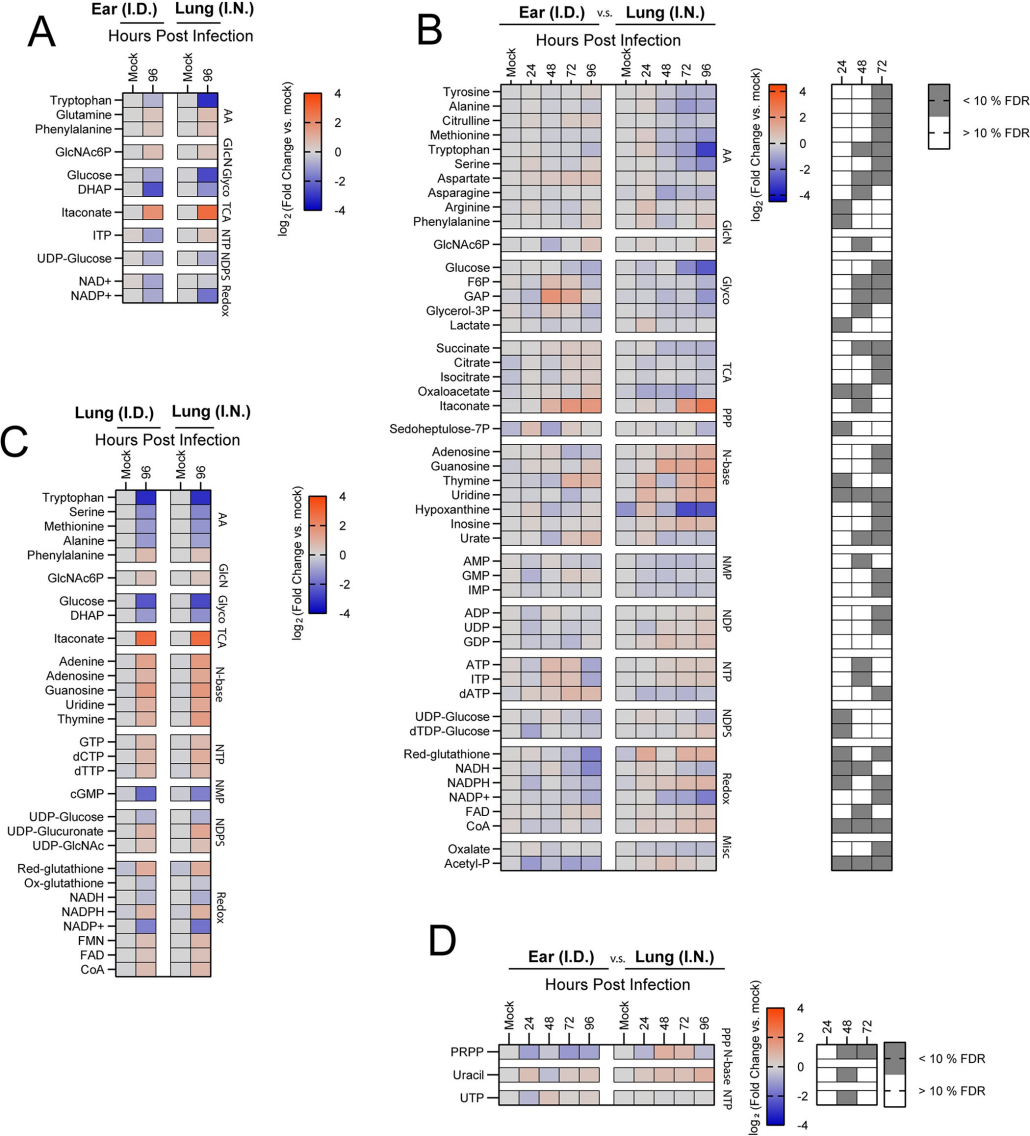
Divergent metabolic responses in the ear and lungs following FTT infection. LC-MS profiling of metabolites in the lungs and ear following i.n. or i.d. infection. (A) Metabolites with conserved changes between the lung and ear 96 hours after infection. (B) Metabolites with significantly divergent patterns between the lung and ear over time. (C) Metabolites with conserved changes between the lungs of i.n or i.d. infected mice 96 hours after infection. (D) Metabolites with significantly divergent patterns between the lungs of i.n. or i.d. infected mice over time. Data are shown as the mean Log_2_FC values relative to uninfected (N = 5 mice per group). Corresponding grey boxes on the accompanying greyscale heatmap indicate significance between i.n. and i.d. infected groups for (B). Statistical significance was calculated using a Benjamini Hochberg test with Q = 10% on log transformed data. For divergent patterns, metabolite hits were identified by comparing magnitude of shift in log_2_FC values between i.n. and i.d. animals at between 48 and 72 hours post infection. For conserved patterns, metabolites were selected through a Benjamini Hochberg test with Q = 10% cutoff using the 96-hour timepoint post infection compared to mock infected controls for both i.n. and i.d. infected groups. Metabolites were binned into families based on structural or network relations which were abbreviated as follows: AA = Amino Acids, GlcN = Gluconeogenesis, Glyco = Glycolysis, TCA = TCA cycle, NTP = Nucleotide Triphosphates, NDP = Nucleotide Diphosphates, NMP = Nucleotide Monophosphates, Redox Metabolism = redox, N-base = Nucleobases, PPP = Pentose Phosphate Pathway, NDPS = Nucleotide Diphosphate Sugars, and Misc = Miscellaneous.

**Fig 5:**
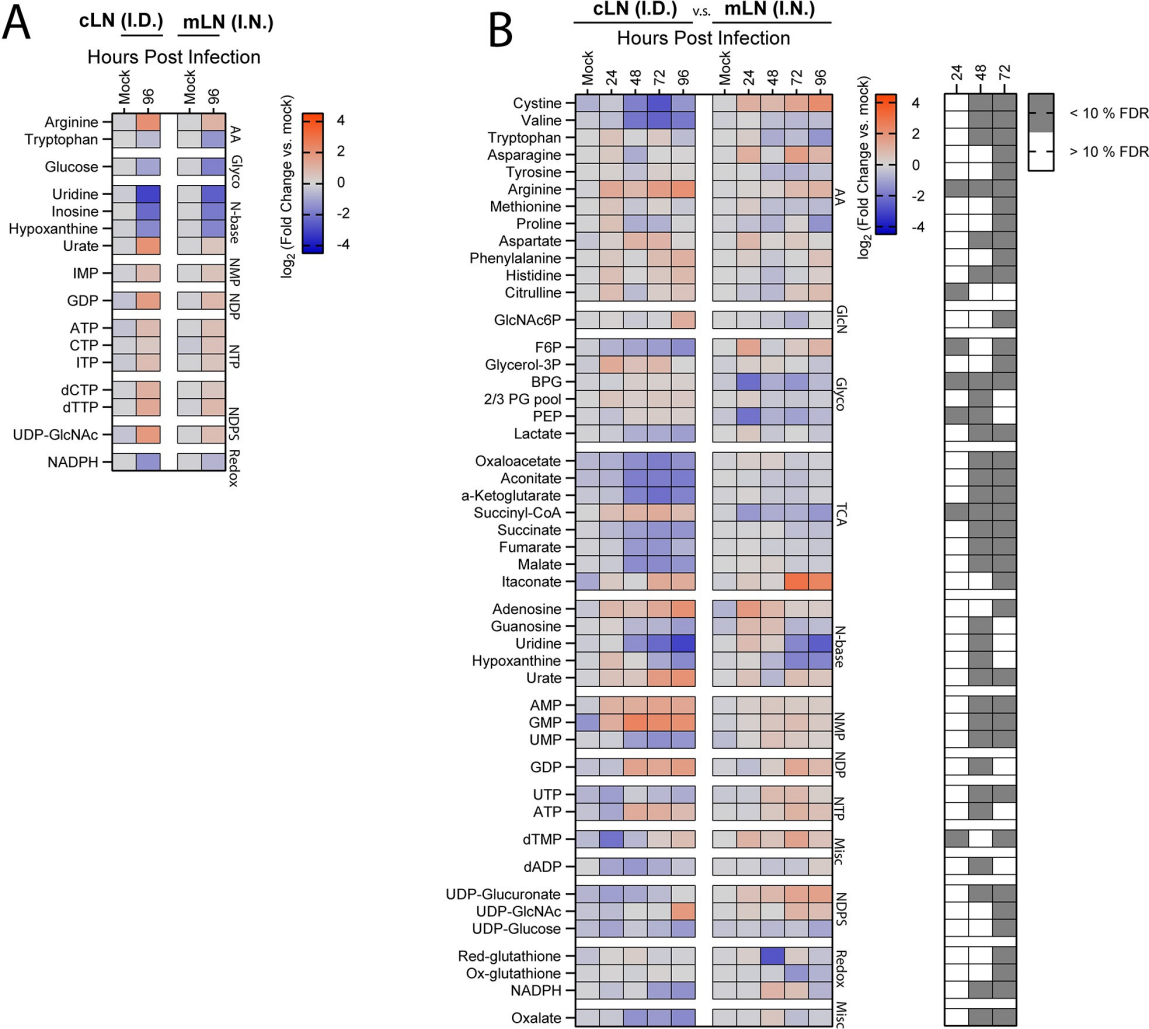
Infection route promotes divergent metabolic programs in the draining lymph node. LC-MS profiling of metabolites in the draining lymph node associated with the primary infection site including the mLN for i.n. infection of the lung, and cLN for i.d. infection of the ear. (A) Metabolites with conserved changes between the mLN and cLN 96 hours after infection. (B) Metabolites with significantly divergent patterns between the cLN and mLN over time. Data are shown as the mean Log_2_FC values relative to uninfected (N = 5 mice per group). Corresponding grey boxes on the accompanying greyscale heatmap indicate significance between i.n. and i.d. infected groups for (B). Statistical significance was calculated using a Benjamini Hochberg test with Q = 10% on log transformed data. For divergent patterns, metabolite hits were identified by comparing magnitude of shift in log_2_FC values between i.n. and i.d. animals at 48 and 72 hours post infection. For conserved patterns, metabolites were selected through a Benjamini-Hochberg test with Q = 10% cutoff using the 96-hour timepoint post infection compared to mock infected controls for both i.n. and i.d. infected groups. Metabolites were binned into families based on structural or network relations which were abbreviated as follows: AA = Amino Acids, GlcN = Gluconeogenesis, Glyco = Glycolysis, TCA = TCA cycle, NTP = Nucleotide Triphosphates, NDP = Nucleotide Diphosphates, NMP = Nucleotide Monophosphates, Redox Metabolism = redox, N-base = Nucleobases, PPP = Pentose Phosphate Pathway, NDPS = Nucleotide Diphosphate Sugars, and Misc = Miscellaneous.

**Fig 6:**
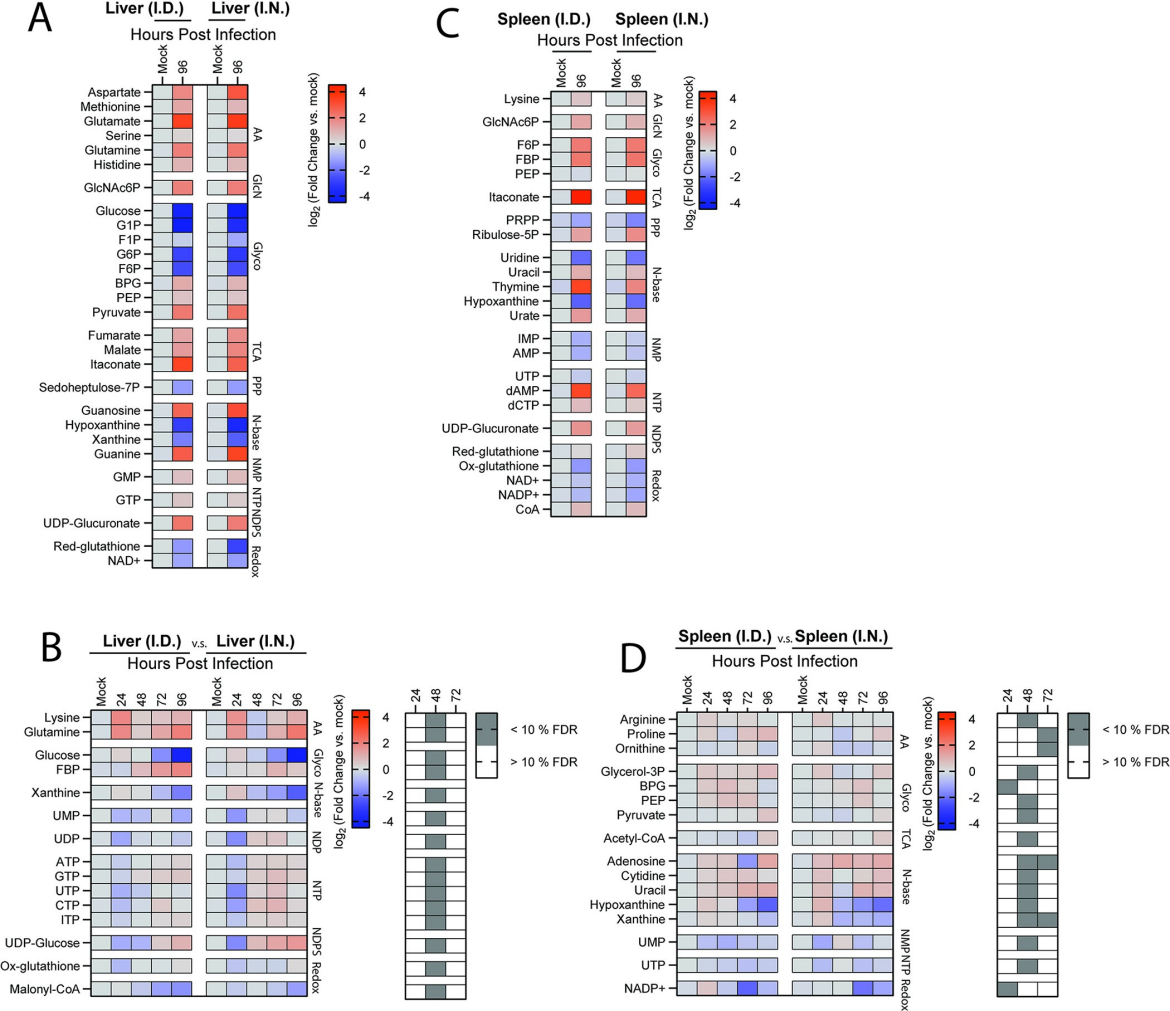
FTT manipulates metabolism of systemic tissues prior to detectable colonization. LC-MS profiling of metabolites in the liver and spleen after i.n or i.d. infection. (A) Metabolites with conserved changes in the liver among i.d. and i.n. infected mice 96 hours after infection. (B) Metabolites with significantly divergent patterns in the liver. (C) Metabolites with conserved changes in the spleen following i.d. and i.n. infected mice 96 hours after infection. (D) Metabolites with significantly divergent patterns in the spleen over time. Data are shown as the mean Log_2_FC values relative to uninfected (N = 5 mice per group). Corresponding grey boxes on the accompanying greyscale heatmap indicate significance between i.n. and i.d. infected groups for (B, D). Statistical significance was calculated using a Benjamini Hochberg test with Q = 10% on log transformed data. For divergent patterns, metabolite hits were identified by comparing magnitude of shift in log_2_FC values between i.n. and i.d. animals at 48 and 72 hours post infection. For conserved patterns, metabolites were selected through a Benjamini-Hochberg test with Q = 10% cutoff using the 96-hour timepoint post infection compared to mock infected controls for both i.n. and i.d. infected groups. Metabolites were binned into families based on structural or network relations which were abbreviated as follows: AA = Amino Acids, GlcN = Gluconeogenesis, Glyco = Glycolysis, TCA = TCA cycle, NTP = Nucleotide Triphosphates, NDP = Nucleotide Diphosphates, NMP = Nucleotide Monophosphates, Redox Metabolism = redox, N-base = Nucleobases, PPP = Pentose Phosphate Pathway, NDPS = Nucleotide Diphosphate Sugars, and Misc = Miscellaneous.

**Fig 7:**
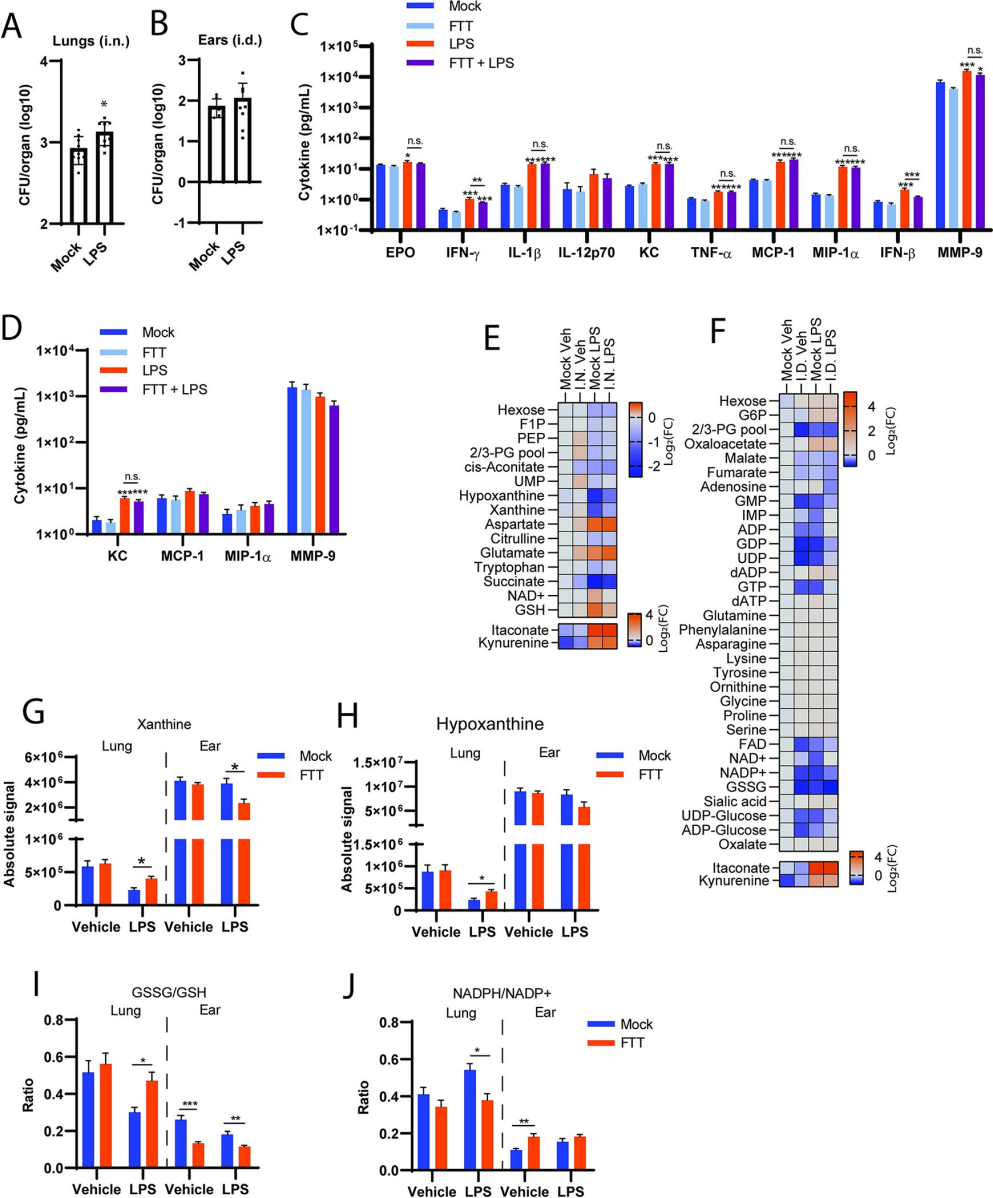
LPS preconditioning of central metabolism enhances FTT replication in the lungs. Mice were preconditioned with LPS 24 hours prior to i.d. or i.n. infection and bacterial burden assessed 24 hours after infection in the ear and lung (A-B). (C) Cytokine/chemokine levels in the lungs. (D) Cytokine/chemokine levels in the ears. (E) Change in metabolite levels (Log_2_FC) relative to mock + vehicle infected controls in the lungs. (F) Change in metabolite levels (Log_2_FC) relative to mock + vehicle infected controls in the ear. (G) Absolute signal of xanthine or (H) hypoxanthine in the lungs or ear as detected by LC-MS. (I) Oxidized/Reduced glutathione ratios in the lungs or ear as detected by LC-MS. (J) NADPH/NADP+ ratios in the lung or ear as detected by LC-MS. Data shown in A-D and G-J are mean ± SEM pooled from two separate experiment (N = 9–14 mice per group). Data shown in E-F are mean Log_2_FC values relative to mock + vehicle treated groups (N = 9–14 mice per group). For (A-B), statistical significance was evaluated using a student’s two-tailed t-test where *p<0.05. For (C-D, G-J), *p<0.05, ***p<0.001 indicates significance relative to mock or between groups where indicated by a line using 2-way ANOVA followed by Tukey’s post-test to correct for multiple comparisons. For (E-F), metabolites hits were identified using a Benjamini Hochberg test with Q = 10% on log transformed data by comparing the magnitude of shift in log_2_FC values between LPS preconditioned and vehicle treated animals.

Statistical significance between group means was determined where indicated using a student’s t-test, or 1- or 2-way ANOVA followed by Tukey’s or Dunnett’s multi-comparison tests correction for false discovery with cut-off levels indicated with data display to compensate for type I error. For metabolomics studies a Benjamini-Hochberg was used. Statistical power was greater than <0.8 to determine sample size, and statistical significance was defined as a probability of type I error occurring at *P* less than 0.05 or a false discovery rate of 10–20% for metabolomic comparisons where indicated. All metabolomic screens (Figs 4–6) were performed with 5 mice per group (N = 5). All other studies (Figs 1–3 and 7) were repeated twice with 4–7 mice per group, then data pooled (N = 9–14 mice total) for statistical analysis. Statistical analysis was performed using GraphPad Prism 9.0, Microsoft Excel, or MarkerView Software 1.3.1 (Sciex).

## Results

### Bacterial replication and dissemination following pulmonary or intradermal infection

To determine how infection dynamics differ after pulmonary or intradermal infection, we first compared mean time to death, bacterial titer, and dissemination kinetics. Mean time to death for i.n. infection was significantly more rapid than i.d. infection (mean = 3.95 days for i.n. versus mean = 4.6 days for i.d. exposure) (Fig 1A). This data was consistent with reports by others in using the Balb/c model [17]. Following i.n. infection, bacteria rapidly replicated in the lung and dissemination to the draining lymph node (mLN), spleen, and liver was evident within 48 hours after infection (Fig 1B–1G). Dissemination to the ear was not detected until 72 hours post infection (Fig 1D). Like the lung, once bacteria seeded these peripheral organs they replicated exponentially (Fig 1B–1G). Twenty-four hours after i.d. infection, bacterial burdens were approximately 10-fold less in the ear than those achieved in the lungs after i.n. infection (Fig 1B). This suggested that the dermal compartment possessed features that did not allow for as optimal replication at early timepoints compared to the lung. However, this limitation was transitory, as the number of bacteria in the ear and lung were similar at later stages of infection (48–96 hours) (Fig 1B–1D). As observed in mice infected i.n., i.d. infected mice had detectable bacteria in the draining lymph node (cLN) and liver within 48 hours of infection which replicated exponentially in these organs following colonization (Fig 1E and 1F). However, we observed a transient and statistically significant difference in bacterial loads in systemic organs that was dependent on the route of infection. Specifically, inoculation with 25 CFU i.n. resulted in significantly higher numbers of bacteria in the corresponding draining lymph node compared to inoculation with 25 CFU i.d. in mice after 48 and 72 hours (Fig 1E). In the liver, i.d. infected animals had significantly higher burdens 48 hours after infection compared to i.n. infected animals (Fig 1F). Interestingly, unlike i.n. infection, i.d. infected mice had detectable bacteria in the spleen within 24 hours after infection which correlated with higher loads at 48 and 72 hours post infection (Fig 1G). Regardless of the time of bacterial colonization or route of inoculation, all organs had the same pathogen burden at the end stage of infection with exception to the ear following i.n. infection (Fig 1B–1F). These data indicated that the lung and draining lymphatics had an increased propensity to support FTT replication at early stages of infection, but this feature did not lead to increased bacterial loads systemically in the livers or spleens that could explain earlier onset of morbidity. Conversely, i.d. infection resulted in less bacterial burdens in the ear and draining lymphatics and was associated with a transient but greater bacterial burden in the spleen and liver after dissemination and a modest delay in onset of morbidity. Therefore, it was plausible that host factors produced early following infection associated with the primary infection site may be influencing disease pathogenesis and the time to onset of morbidity.

### Comparative analysis of the inflammatory response following intranasal or intradermal infection

Differences in timing and/or magnitude of the host inflammatory response may contribute to increased replication, lymph node colonization, and time to morbidity of FTT after pulmonary versus intradermal infection. To examine this relationship, we performed a spatiotemporal analysis of several cytokine/chemokines across critically involved tissues with respect to each infection route. A biphasic pattern occurred with limited cytokine production between 24–48 hours post infection and extensive cytokine production between 48–96 hours post infection, consistent with sepsis and onset of morbidity (S1 Fig). Because of this biphasic response, we focused our analysis on the 24–48-hour timeframe to identify early factors that may correlate with increased virulence after i.n. exposure (Fig 2A–2E). Cytokine levels in the ear and cLN after i.n. infection was below the detection limit during this early timeframe (uninfected, 24–48 hours post infection) (S2 Fig), consistent with the absence of bacteria (Fig 1C). Increased IFN-γ, IL-6, and KC levels occurred in the lungs by 48 hours post infection after i.n. compared to i.d. infection consistent with higher bacterial loads in this tissue following i.n. infection (Fig 2A). However, with the exception of increased KC and a transient increase in IL-12p40 levels, no evidence of change in systemic markers of inflammation, occurred in the liver following i.n. infection within the first 48 hours (Fig 2C). In contrast to the correlation of the relative bacterial load with production of cytokine observed in the lungs and ears, route of infection greatly, and disparately, influenced the ensuing inflammatory response in the respective draining lymph nodes, i.e. mediastinal lymph node (mLN) for the lung and the cervical lymph node (cLN) for the ear. Specifically, i.n. infected animals had bacterial loads in the mLN that were greater than those detected in the draining cLN of i.d. infected mice at 48 hours (Fig 1D). However, increased bacterial burden did not translate into increased production of IFN-ɣ, IL-6, or TNF-α in the mLN compared to uninfected controls at this time point. Further, KC, MCP-1, MIP-1α, and RANTES in the draining mLN of i.n. infected animals were decreased compared to uninfected mice (Fig 2B). In contrast to i.n. infected animals, mice that were inoculated i.d. had significantly greater concentrations of both RANTES and TNF-α in the draining cLN compared to uninfected controls including at a time point in which bacteria were not detected in this tissue (24 hours after infection). This suggested that reprogramming of peripheral lymph tissue following infection was differentially regulated prior to bacterial colonization and was dependent on the route of infection. We hypothesized that the increased responses observed in the lymph nodes of i.d. infected animals may corelate with heightened production of cytokines in the ears of these animals within the first 24 hours of infection. Indeed, i.d. infection resulted in significantly greater concentrations of IL-12p40 and a trend of increased production of other cytokines assessed in the ear 24 hours after infection compared to uninfected controls (Fig 2A–2E). Moreover, by 48 hours after infection every cytokine assayed was significantly increased in the ear after i.d. infection in contrast to just IFN-ɣ and IL-6 in the lung after i.n. infection (Fig 2A and 2E). Together these data suggests that i.d. infection promotes an earlier local and systemic inflammatory response than i.n. infection that was dissociated with relative bacterial load.

During the sepsis stage of disease (72–96 hours), i.n. infection continued to promote greater inflammation at the site of inoculation, i.e. the lungs, relative to i.d. infection (S1A Fig). Intradermal infection promoted comparable cytokine production in the ear (S1B Fig). In the draining lymphatics, except for modest but significant differences in IL-6 and KC, concentrations of cytokines were similar regardless of the route of infection (S1C Fig). In the liver and spleen, a divergent temporal pattern in cytokine production was evident with i.d. infection. Specifically, increased responses in IL-6, KC, and MCP-1 were evident at 72 hours post infection, which were then reversed at 96 hours post infection with i.n. exposure promoting the greater response (S1D–1E Fig). Together, these data suggested that infection route informs transient differences in the magnitude of the inflammatory responses, with i.d. infection promoting earlier inflammation in the draining lymph nodes and a modestly reduced response at later stages of infection in the lungs relative to i.n. infection.

In addition to microbial products, dead and dying host cells can also trigger inflammatory responses. Since we observed differences in cytokine production that did not correlate with bacterial loads in some tissues, we next assessed the burden of dead and dying cells following infection. The flipping of phosphatidylserine (PS) to the outer leaflets of membranes of dead or dying cells increases binding and uptake by resident macrophages [18] and detection of this lipid on the surface of cells can be used as an additional biomarker of inflammation, tissue damage, and cell death (S3A–S3F Fig) [19]. Therefore, we next examined the uptake and tissue distribution of the far-red fluorescent annexin V derivative AnnexinVivo-750 which binds to surface exposed PS. Intradermal infection resulted in increased AnnexinVivo 750 uptake in the draining cLN as early as 48 hours post infection consistent with an earlier inflammatory response (S3E Fig). In the ear, increased AnnexinVivo 750 signal was not evident until 72 hours post infection, which was 24 hours earlier than when it presented within the lungs (Figs S3F and 2C). No significant difference in AnnexinVivo 750 uptake was evident in the liver, lungs, or spleen between i.d. or i.n. infected mice during the first 48 hours. However, by 72 hours post infection significant inflammation and/or cellular damage occurred in the spleens of infected mice, irrespective of infection route, as indicated by increased AnnexinVivo 750 uptake (S3D Fig). Finally, we observed significantly increased AnnexinVivo 750 uptake at 96 hours post infection in all tissues assessed consistent with acute onset tissue damage and morbidity (S3A–S3F Fig). These data further strengthen the conclusion that i.d. infection results in earlier inflammation and/or tissue damage in the draining lymphatics and ear relative to the pulmonary compartment after i.n. infection.

### Glucose uptake and distribution following FTT infection

Shifts in host metabolism can be an essential event in the developing inflammatory response with early upregulation of glycolysis supporting rapid activation of host inflammatory programs [9]. The distinct spatiotemporal patterns in cytokine production between intradermal and intranasal infection implicate potential differences in the propensity to increase glycolysis after FTT infection. Measuring distribution and uptake of glucose derivatives with preclinical imaging techniques can identify tissues where increased glycolysis is actively supporting inflammatory functions [19,20]. Therefore, we next assessed the tissue distribution of the glucose derivative XenoLight RediJect 2-DG 750 (RJ2-DG-750) using optical fluorescence imaging in mice following i.n. or i.d. infection. A transient increase (p = 0.055) in RJ2-DG-750 uptake in i.d. versus i.n. infected mice was evident 24 hours following infection in the shaved thorax region of the mice, which included signal from the skin and most major internal organ systems (Fig 3A). However, we could not distinguish which tissues were predominantly responsible for increased signal using whole animal imaging approaches. Therefore, tissues were excised and examined individually ex vivo to delineate whether i.d. or i.n. infection caused tissue specific variations in RJ2-DG-750 uptake over time. Consistent with whole body imaging, irrespective of infection route and consistent with onset of morbidity the predominant glycolytic response occurred by 96 hours post infection in all tissues assessed, (Fig 3B–3F). Interestingly, neither the ear nor lung increased RJ-2DG uptake until later stages of infection, suggesting the metabolic potential of the primary infection site is manipulated by FTT to limit uptake of glucose or refractory to rapid upregulation and, at least at the site of infection in the ear, is dissociated from production of pro-inflammatory cytokines within the first 48 hours of infection (Fig 3B and 3C). Consistent with this notion, (although not statistically significant) both the ear and lung appeared to have reduce RJ2-DG-750 levels at 24 hours post infection relative to mock infected controls (Fig 3B and 3C). Conversely, i.d. infection promoted earlier upregulation of glycolysis in the draining cLN relative to the mLN following i.n. infection. This suggested that while the ear may be refractory to upregulate RJ2-DG-750 uptake, the host is responding rapidly at the level of the draining cLN (Fig 3D).

At later stages of disease, temporal differences were observed in systemic tissues. We observed earlier uptake of RJ-2DG in the liver and increased uptake in the spleen at 96 hours post i.n. inoculation compared to the same tissues following i.d. inoculation (Fig 3E and 3F). Together, these data indicated that i.d. infection promoted a substantive earlier glucose uptake response consistent with earlier cytokine production. However, at later stages of disease i.n. infection drove increased glucose uptake in the liver and enhanced levels in the spleen, indicative of an exacerbated metabolic response in the periphery.

### Divergence in central metabolism in the ear and lungs following FTT infection

Our data above suggested that metabolic flux and associated host metabolites may be differently regulated following i.n. and i.d. infection. Therefore, we next characterized changes in a wide range of host metabolites associated with distinct infection routes to identify pathways that may delineate common or divergent changes within or between tissues over time. We used two analytical approaches to curate this data to identify both conserved or divergent responses between infection route in primary exposed tissues (i.e. lungs versus the ear) and systemic tissues (i.e. draining lymphatics, spleen, and liver). For conserved responses, a Benjamini-Hochberg correction with a false discovery rate (FDR) set at 10% comparing mock infected controls to 96 hours after infection was employed. For divergent responses, a Benjamini-Hochberg correction for FDR set at 10% was used to identify significant differences in Log_2_(Fold Change) values of metabolites between tissues at 24, 48, and 72 hours post infection. Finally, we categorically binned metabolites that passed FDR cut-offs into families based on their structural relationship (i.e. amino acids) or by association with metabolic pathways (i.e. glycolysis, TCA cycle, redox reactions). This allowed for mapping isolated changes in specific metabolites within a network or involvement of the entire network.

We first compared the ear and lungs after i.d. or i.n. infection, respectively. Conserved changes included decreases in the amino acid tryptophan in the ear and lung albeit to different magnitudes, whereas glutamine and phenylalanine increased in both tissues (Fig 4A). Itaconate, a well-defined marker of TCA cycle reprogramming involved in increased inflammatory cascades, was elevated in both infection sites, but detectable increases were evident 24 hour earlier in the ear than in the lung [21] (Fig 4B). Consistent with increased glycolysis at the end stage of infection observed via RJ-2DG uptake, decreased glucose and dihydroxyacetone phosphate (DHAP) levels, were evident indicative of increased flux in both tissues (Fig 4A). Finally, NAD+ and NADP+ levels progressively decreased in both tissues, potentially indicative of increased bioenergetic demand (Fig 4B). In contrast to these conserved signatures, we observed more metabolites that significantly diverged between the ear and lung over time. Among divergent responses, i.n. infection resulted in a transient increase in arginine and phenylalanine at 24 hours post infection in the lungs followed by a general depletion in amino acids, an effect that was absent in the ears of i.d. infected mice (Fig 4B). Additionally, a depletion or reduction in glycolytic intermediates, nucleotide monophosphates (NMP), dATP, and TCA cycle metabolites occurred over time and was unique to the lungs among i.n. infected mice compared to the ear of i.d. infected mice (Fig 4B). Finally, i.n. infection resulted in an increase in most nucleobases except for hypoxanthine, which was significantly depleted at later stages of infection. The divergence in metabolic programs between ear and lung also included dysregulation in several redox or bioenergetic intermediates (Figs 4B and S4–S6). Specifically, we observed statistically significant divergence in the NADPH/NADP+ levels in the lungs of i.n. infected mice relative to the ear of i.d. infected mice (S4A Fig). The increased NADPH/NADP+ ratio in the lungs was not paralleled by increased NADH/NAD+ ratio or reduced/oxidized glutathione ratio. This suggested that the response was a function of pentose phosphate pathway regulation rather than oxidative stress or nucleotide salvage (S4F and S5A Figs). We also observed a reduction in the AMP/ATP ratio in the lungs of i.n. infected mice relative to ear after i.d. infection at 48 hours, indicative of an increased bioenergetic potential (S6F Fig). The combination of increased NADPH/NADP+ ratio and bioenergetic potential are conducive of increased anabolic functions, including fatty acid synthesis which has been reported to be an energy source for FTT replication [22,23]. Conversely, the ear had higher baseline reduced/oxidized glutathione and NADH/NAD+ ratios that decreased with time, suggesting elevated redox stress (S4F and S5A Figs). Ultimately, these data support that the intradermal compartment undergoes earlier increased glycolytic and oxidative stress responses relative to the lungs, and generally has less depletion of metabolites during disease pathogenesis.

To determine if anatomically distinct infection routes drove divergent responses in the lungs, we next compared the metabolic response of only the lungs after i.n. or i.d. infection over time. Surprisingly, metabolic changes were highly conserved (Fig 4C and 4D). There were no significant differences in NADPH/NADP+ and NADH/NAD+ ratios over time (S4B and S4G Fig). Changes in reduced/oxidized glutathione ratios, ADP/ATP and AMP/ATP ratios were markedly conserved, with significant, but transient differences observed at 24 and 48 hours, respectively (S5B and S6B Figs). Further, 29 metabolites of the 111 metabolites detected shared common directional change between infection routes, including several amino acids, glucose, DHAP, itaconate, several nucleotide/nucleoside bases, nucleotide triphosphates, and cGMP, metabolites associated with redox status, and nucleotide diphosphate sugars (Fig 4C). Conversely, only 3 metabolites Phosphoribosyl pyrophosphate (PRPP), Uracil, and UTP) passed 10% FDR cutoff for differential change, and differences were marginal or transient (Fig 4D). Therefore, the metabolic response to infection in the lung was remarkably conserved regardless of where infection was first established.

### Divergence in central carbon metabolism in the draining lymph nodes after FTT infection

Significant differences in glucose uptake (Fig 3D) and inflammation (Fig 2) observed in the cLN after i.d. infection compared to the mLN after i.n. infection potentially indicated broader divergence in the local metabolic response defining the response in these tissues. Therefore, we next compared the metabolic profiles of the respective draining lymph nodes associated with i.n. and i.d. infection sites. (Fig 3D). Conserved metabolic responses included changes in isolated amino acid, glycolytic, nucleobases, and mono/di/tri nucleotide phosphates. We observed depletion of tryptophan, glucose, and nucleobases whereas there were accumulations of arginine, urate mono, di and triphosphates in both the cLN and mLN over time (Fig 5A). Conversely, divergent responses identified included depletion in cystine in the cLN after i.d infection and accumulation of this metabolite mLN after i.n. infection (Fig 5B). Additionally, arginine and citrulline increased earlier in the cLN compared to the mLN. Divergent patterns in glycolysis were also evident, including decreased fructose-6-phosphate in cLN versus increased levels in the mLN over time. The mLN also contained reduced downstream glycolytic intermediates bisphosphoglyceric acid (BPG) and PEP relative to cLN (Fig 5B). Although it did not meet the <10% FDR, we observed a transient increase in itaconate in the cLN 24 hours after infection which then fluctuated over time. Itaconate accumulation occurred at 72 hours post infection in both the cLN and mLN, however responsiveness was significantly enhanced in the mLN relative to the cLN at this time point (Fig 5B). Intradermal infection resulted in depletion of TCA cycle intermediates in the cLN, apart from succinyl-CoA which increased with time (Fig 5B). Succinyl-CoA ligase is responsible for converting Succinyl-CoA to succinate, generating either ATP or GTP in the process, and this process can occur under oxygen deficient conditions [24]. Therefore, accumulation of Succinyl-CoA and cofactor GDP may be indicative of dysregulated Succinyl-CoA ligase activity in the cLN. Inversely, Succinyl-CoA was depleted in the mLN (Fig 5B). Increased AMP and GMP further suggest significant alteration in nutrient stress in the cLN relative to the mLN. cLN also had higher baseline reduce/oxidized glutathione ratio compared to the mLN, indicative of increased redox activity (S5C Fig). Together, these data support greater metabolic responsiveness in the cLN after i.d. infection that were supportive of pro-inflammatory activity. In contrast, the mLN demonstrated less dramatic metabolic dysregulation consistent with a more inflammatory null phenotype, parallel to the lung, at earlier stages of infection.

### FTT manipulates metabolism of systemic tissues prior to detectable colonization

Our data demonstrated the metabolic response in the lungs was remarkably conserved irrespective of infection route (Fig 4). However, the liver and spleen also become significantly involved in disease pathogenesis, and functional failure of these tissues has been attributed to onset of morbidity [25]. If or how infection route informs the host metabolic response in the liver and spleen has not been explored. Therefore, we evaluated changes in the metabolic milieu in the spleen and liver over time following i.n. and i.d. infection. Like the lungs, metabolic changes in the liver were highly conserved between infection routes (Fig 6A). Differences between i.d. and i.n. infection route in the liver occurred only at the 48-hour timepoint post infection and were associated with magnitude of change but not change of the trends over time (Fig 6B). Generally, i.n. infection induced a more augmented metabolic response between 24–48 hours post infection compared to i.d. exposure. This observation was surprising, since we did not detect bacterial burden within the liver at this time point. This suggested either host factors or potentially bacterial effectors produced in other organs are driving metabolic responses at distal sites. The early responses observed prior to bacterial colonization included increases in several amino acids (Methionine, Glutamate, Serine, Histidine), and decreased glycolytic intermediates. This pattern indicated potential activation of glycolytic or gluconeogenic pathways. Upon bacterial colonization of the liver, disease progression was characterized by accumulation of amino acids, increased downstream glycolytic intermediates, and reprogramming of the TCA cycle including itaconate production. At the latest stages of disease, we observed a loss of xanthine/hypoxanthine, loss of reduced glutathione and NAD+ levels, and alterations in the ADP/ATP and AMP/ATP ratios. Together these changes suggested dysregulation of the redox balance and/or bioenergetic deficits that were consistent with severe tissue damage (S5D, S6D and S6I Figs).

Assessment of changes of metabolite levels in the spleen followed a similar pattern to the liver. The metabolic responses to infection were highly conserved regardless of infection route (Figs 6C, 6D, S4E, S4J, S5E, S6E and S6J). Further like the liver, many metabolites had measurable changes as early as 24 hours post infection which is before detectable colonization of this organ following i.n. infection. Conversely, 16 out of the 111 metabolites detected diverged between i.d. and i.n. infection, but like the liver this pattern only occurred at 48 hours post infection (Fig 6D). Furthermore, significant differences were primarily due to an augmented, but transitory, response in the i.n. infected group. Notably, we observed similar accumulation of glycolytic intermediates and dysregulation in the pentose phosphate pathway, hypoxanthine/urate metabolism, and redox networks. Together, these data suggested that early after infection the site of inoculation influenced metabolic processes at distal sites and i.n. infection often resulted in a more marked reversal of metabolite trends between 24 and 48 hours post infection relative to i.d. inoculation. However, once peripheral tissues were colonized these differences were resolved with the liver and spleen entering an energetic crisis at late stages of disease progression associated with organ failure and onset of morbidity.

### LPS preconditioning of central metabolism enhances FTT replication in the lungs

As shown in the above metabolic screen, i.n. infection results in the early reprogramming of pulmonary and lymph node tissues that are consistent with poor/low inflammatory state, e.g. minimal evidence of a glycolytic response, despite the presence of rapidly replicating bacteria. We hypothesized that one could reverse this phenomenon by infecting hosts in which the pulmonary environment was in a hyper-inflamed state resulting in either superior control of infection or extended mean time to death as observed in animals infected with FTT intradermally. LPS exposure is known to drive increased glycolysis and TCA cycle reprogramming [8,26]. Therefore, we exposed mice to a sublethal dose of LPS prior to i.n. infection to establish a glycolytic environment in the lung. Twenty-four hours after intraperitoneal (i.p.) injection, statistically significant increases in RJ-2DG were observed in the pulmonary compartment of LPS treated mice compared vehicle treated controls confirming establishment of a more glycolytic environment (S7A Fig) No measurable uptake of RJ2-DG-750 occurred in the ear after i.p. LPS exposure, possibly due to reduced vascular perfusion relative to the lungs (S7B Fig). Twenty-four hours after LPS-preconditioning, mice were infected either i.d. or i.n. and markers of inflammation or carbon metabolism were examined after an additional 24-hour period in the lungs or ear, respectively. Surprisingly, LPS preconditioning resulted in significant increases in FTT burdens in the lungs after i.n. infection, but not in the ear after i.d. infection among LPS preconditioned mice (Fig 7A and 7B). Consistent with a pro-inflammatory state, LPS-preconditioning significantly increased EPO, IFN-γ, IL-1β, KC, TNF-α, MCP-1, MIP-1α, and IFN-β levels in the lungs (Fig 7C) and pulmonary FTT infection significantly reduced IFN-γ, IFN-β, and MMP9 levels among LPS preconditioned mice. This interference with LPS driven cytokine production was consistent with prior findings in which LPS delivered after FTT infection resulted in a dampened inflammatory response in the lung [5]. We next analyzed the same cytokine/chemokine panel in the ears of i.d. infected mice preconditioned with LPS. Most of the cytokine/chemokine levels were below detection limits except for KC, MCP-1, MIP-1α, and MMP-9, implicating the intradermal response was more limited following a systemic LPS exposure (Fig 7D). However, LPS preconditioning significantly increased KC, indicative of early chemotaxis activity in the ear or associated vasculature (Fig 7D). Intradermal infection had no effect on MCP-1, MIP-1α, or KC levels. However, similar to the lung following i.n. infection MMP-9 levels trended downward (p<0.24) within the ear of FTT infected mice with LPS preconditioning relative to mock control (Fig 7D).

We next examined if FTT manipulated host metabolic milieu following LPS preconditioning. Consistent with LPS driven glycolytic responses, metabolites associated with glycolysis as well as increased itaconate and kynurenine, hallmark signatures supportive of host innate immune function, were modulated by LPS exposure [21,27]. Intranasal FTT infection modestly reversed patterns in Fructose-1-Phosphate, ph (F1P), Phosphoenolpyruvic acid (PEP), and 2,3-Bisphophoglycerate (2/3-PG) in LPS-preconditioned mice, indicative of direct manipulation of canonical glycolytic flux during pulmonary FTT infection (Fig 7E). FTT infection also significantly reversed LPS-induced losses of hypoxanthine and xanthine, which have been implicated in the development of acute lung injury (Fig 7E, 7G and 7H) [16,28,29]. Finally, i.n. FTT infection reversed LPS-induced effects on redox metabolism through NAD+ and reduced glutathione (GSH) levels (Fig 7E). Further examination of cellular redox status indicated i.n. infection significantly reversed LPS-preconditioning effects on oxidized/reduced glutathione ratios (Fig 7I). Following i.d. infection and similar to the lung, itaconate and kynurenine were increased with LPS exposure, supportive of a pro-inflammatory program in the ear despite the low uptake of RJ2-DG-750 observed (Figs 7F and S6). However, unlike i.n. infection the overall response to i.d. infection or LPS-preconditioning alone were markedly similar. For example, apart from NAD+, IMP, oxaloacetate, G6P, itaconate, and kynurenine vehicle preconditioning plus i.d. infection induced changes in metabolite levels equivalent to LPS-preconditioning with mock infection (Fig 7F). FTT infection decreased the oxidized to reduced glutathione ratio in the ear similar to LPS-preconditioning and promoted a significant loss in xanthine levels, and a trend in the loss of hypoxanthine (p = 0.07, Fig 7F–7I). Despite the high similarity between i.d. infection and LPS preconditioning, FTT was successful at reversing several metabolite patterns among LPS-preconditioned mice in the ear’s dermal compartment. Specifically, FTT infection reversed LPS-preconditioning impacts on purine metabolism (i.e., ADP, GDP, GMP) and several bioenergetic intermediates (FAD, NAD+, and NADP+) (Fig 7F). Further examination of the NADH/NAD+ ratio ratios revealed no significant dysregulation despite the loss of NAD+ (S7C Fig). A comparative analysis of the NADPH/NADP+ ratio revealed striking differences between the lungs and ear after i.n. or i.d. infection. Specifically, FTT infection corrected an elevated NADPH/NADP+ ratio in the lungs of LPS-preconditioned mice, whereas in the ear, FTT infection did not correct the NADPH/NADP+ ratio (Fig 7J). Rescue of NADPH/NADP+ ratios in the lungs may be associated with FTT manipulation of the pentose phosphate pathway or NADPH oxidase response. Conversely, the increased NADPH/NADP+ ratio in the ear after FTT infection, coupled with the dysregulated oxidized/reduced glutathione ratio (GSSG/GSH) measured (Fig 7I), pointed towards activation of the host innate redox response. Together, these data underscore that the overall differences in how FTT manipulates host metabolism in the lungs or ear to influence disease progression are modest and primarily influence the timing of the response. Further, our data also show the ability of FTT to reverse established metabolic states to a more favorable environment for replication and this is dependent on the site of infection. Specifically, these data support that the metabolic niche of the lungs was more supportive of enhanced FTT replication and involved manipulation of both the pentose phosphate and redox metabolism as elements of enhanced disease pathogenesis.

## Discussion

The relationship between exposure route and divergent mortality rates following FTT infection has been a long-standing enigma [3,4]. The propensity of a tissue’s central metabolic milieu to be exploited by FTT or undergo rapid metabolic reprogramming to elevate immune barriers, is a potential factor contributing to this difference. In mice, which are highly sensitive to FTT, differences in mortality rates are far less evident and are primarily associated with changes in mean-time-to-death (Fig 1A). However, since delivery of the same low dose inoculum, e.g. 25 CFU, via either route is uniformly lethal, this feature provided a unique strength in uncovering differential tissue responses to infection and how that influenced early metabolic changes in the periphery prior to detectable bacterial colonization. In the current study, through comparison of pulmonary and intradermal infection routes we demonstrated that the host metabolic response was highly dynamic over the progression of infection, ultimately concluding in a dysregulated state with onset of morbidity. The overall trajectory of the host metabolic response towards dysregulation was remarkably conserved within the lungs and systemic organ systems irrespective of exposure route, suggesting FTT is exquisitely adept at manipulating diverse metabolic environments into a state conducive for dissemination and replication. However, early delays in metabolic reprogramming were evident in the lungs and draining lymph nodes after pulmonary exposure compared to the local tissue and associated lymph nodes following i.d. infection. The delay in metabolic shifts were associated with greater bacterial replication in the lung within the first 24 hours compared to the dermis. Furthermore, pulmonary infection manifested an augmented systemic response in several metabolic networks and inflammatory cytokine profiles after dissemination. These observations were consistent with the acute onset of severe disease after pulmonary FTT exposure. Furthermore, we demonstrated that FTT was particularly adept in altering lung metabolism even under extrinsic metabolic pressures such as those promoted by systemic LPS exposure. This suggested the pulmonary compartment had greater inherent susceptibility to exploitation relative to intradermal compartment at early stages of infection.

Rapid reprogramming of carbon metabolism is required for activation of innate immune functions needed to control infection [8]. However, anatomically distinct tissues do not share the same metabolic milieu that hypothetically could influence their ability to rapidly respond. Following inhalation, FTT initially established infection in the lung, predominantly in alveolar macrophages (AM) [30]. The airway lumen is an environment that consists of low levels of extracellular carbon sources needed to drive inflammatory responses. For example, the airway lumen’s glucose concentrations are less than 10% of the concentration found in the blood [31]. Additionally, apart from alanine it has been reported that glucogenic and ketogenic amino acids are mostly absent in the lower airways [31]. Alveolar macrophages and airway epithelial cells are bathed in surfactant which, through both physical inhibitory activity and catabolic products, can limit host inflammatory programs [31,32]. Finally, it has been reported that under normal conditions the resident alveolar macrophages in the airways, which are likely an early reservoir for FTT, are preferentially in a state of oxidative phosphorylation for energy production [30,33]. The combination of these basal conditions is likely to have contributed to protected replicative niche during the early stages of infection with FTT. Consistent with this notion, FTT exploited host metabolism in the pulmonary compartment at early stages to a greater extent than the intradermal compartment resulting in initially higher bacterial titers in this tissue compared to the dermal compartment (Figs 1 and 4). Rate of expansion slowed between 72–96 hours post infection in the lungs, corresponding with increased cell death (Figs 1 and S2). Like the lungs, the skin also preferentially utilizes OXPHOS [34]. This background phenotype may make this location initially accommodating to FTT. However, intradermal sampling has shown glucose availability to be similar to levels present in the arterial blood, suggesting unrestricted access to carbon sources that support increased inflammation [35]. The skin also can rapidly increase transporters to access inflammation supporting amino acids [34]. Therefore, upon perturbation of dermal barrier and detection of pathogens or danger signals associated with disruption of the dermal barrier, rapid access to carbon sources in the blood could accelerate the host inflammatory response to FTT infection.

An alternative explanation for early differences in temporal dynamics in the host response to FTT infection between the lungs and intradermal compartment could be a function of divergent baseline redox potential. In the ear, redox programs (NADH/NAD+, oxidized/reduced glutathione) started with higher baselines and responded dynamically after infection whereas the redox programs in the lungs were tightly maintained at a lower baseline activity (S3 and S4 Figs). Consequently, the redox stress initially encountered by FTT would be limited in the lungs allowing for greater replication. Conversely, FTT within the ear would encounter increased redox stress resulting in slower replication, cell death, and/or unmasking of pathogen associated molecular patterns that in turn may trigger earlier inflammatory cascades. Additionally, changes in redox dysregulation alone could potentiate inflammation in the ear and draining lymphatics.

In addition to evading host inflammatory responses, FTT must compete for available intracellular carbon sources to successfully replicate. A convenient virulence strategy would be to manipulate host metabolic hubs to achieve both requirements. For example, FTT is reported to primarily rely on host amino acids, fatty acids, and glycerol for replication [22,23,36]. Coincidently, maintaining mitochondrial function, limiting pro-inflammatory glycolysis, and increasing cellular bioenergetics could meet both demands [12,15]. Our data presented features in both the ear and lung consistent with this notion, however greater evidence for host manipulation for a nutrient advantage was present in the lungs. We currently do not know the absolute availability of intracellular carbon sources for FTT metabolism between the lungs and ear. A comparative analysis will require a reliable normalization factor as well as more detailed understanding of the metabolic intracellular microenvironment in which FTT resides early after infection. We would predict these niches to be different, as it has been reported with *F*. *tularensis holarctica* LVS that alveolar macrophages, which as stated above reside in a nutrient restricted environment, are primarily targeted early in the lung. However, Langerhans cells are initially targeted in the dermal compartment by *F*. *tularensis holarctica* LVS, and these cells potentially have increased access to inflammation supporting metabolites as well as facilitate earlier conditioning of the draining lymphatics upon migration [37]. Despite this technical hurdle, several conclusions can be inferred based on dynamic changes between whole organ/tissues. For example, increased amino acid depletion was evident in the pulmonary compartment relative to the intradermal compartment over time, potentially indicating increased amino acid consumption by FTT (Fig 4A). Additionally, a high NADPH/NADP+ ratio, which occurred in the lungs after FTT infection, could be a manifestation of either increased pentose phosphate pathway flux and/or lipogenesis, both of which provide essential fuel sources [22,23,36,38]. The observation that FTT maintains or limits modulation of the NADPH/NADP+ ratio and pentose phosphate pathway in the lungs even under extrinsic metabolic pressures provided by preconditioning with LPS further support the involvement of these networks. Together, these data suggested that the lungs, relative to the intradermal compartment, provided a more favorable nutrient environment with respect to carbon sources preferentially catabolized by FTT.

Historically the lungs have been characterized as an “immune tolerant” tissue due two low responsiveness to environmental exposures, a feature that is likely exploited by FTT [39]. Placing the pulmonary compartment under a metabolic stressor, such as LPS, disrupted this basal suppressed phenotype as evidenced by increased cytokine/chemokine production and metabolic programs consistent with a pro-inflammatory environment (Fig 7). We initially hypothesized that when FTT would encounter this environment, replication would be restricted, and metabolic pathways required for virulence could be extrapolated. Surprisingly, we observed the opposite outcome wherein LPS preconditioning facilitated even greater FTT replication in the lungs compared to vehicle controls (Fig 7). FTT infection specifically reversed metabolite levels associated with end-stage glycolytic flux, potentially indicating LPS-stimulated uptake of glucose was being re-directed towards other catabolic pathways. Redirecting glucose towards other networks can result in both limited inflammation and/or provide additional fuel sources, such as through the pentose phosphate pathway or glycerol production, for FTT replication. FTT infection also increased intracellular aspartate and glutamate levels in the presence of LPS preconditioning. These amino acids are specifically connected via the aspartate/malate shunt and constitutes a major source for nitrogen assimilation for mammalian cells and for mitochondrial respiration [40]. Therefore, increasing host nitrogen assimilation and glutaminolysis for mitochondrial respiration could support increased FTT replication. Future studies by our laboratory will address dependence of enhanced FTT replication in LPS-precondition mice on these identified pathways through nutrient restriction and/or enzymatic inhibitory approaches.

One limitation in interpreting results from this study was uncoupling metabolic programs FTT affected to limit LPS-induced injury, and which metabolic programs were being exploited for hyper-replication. Systemic LPS exposure is known to not only drive pro-inflammatory metabolic reprogramming but also acute lung injury [41]. FTT contains both capsule and lipid effectors that can limit host cell death and inflammation [11,12,15,42], and thereby may also reduce acute lung injury induced by systemic LPS exposure. Dysregulation in xanthine/hypoxanthine has been reported to be a positive biomarker of acute lung injury [43]. Xanthine and hypoxanthine are integral to the nucleotide salvage pathway and uric acid cycle. Within the uric acid cycle, xanthine oxidase can utilize hypoxanthine to generate urea and ROS. Consequently, FTT-associated reversal in hypoxanthine/xanthine levels likely reflects recovery or protection against redox activity and/or tissue damage responses resulting from LPS preconditioning. Consistent with this notion, FTT also reversed dysregulation in oxidized to reduced glutathione levels after LPS preconditioning, suggesting limitation on lung ROS production. It is not known if nucleotide salvage pathways in the host can be exploited by FTT for increased nutrient sources acquisition. However, increased nucleotide salvage via hypoxanthine is reported to facilitate increased tissue viability in several non-infectious models by increasing ATP levels, and thereby could indirectly support enhanced FTT infection by maintaining the replicative intracellular niche [44].

Perhaps the most surprising observation in this study was the ability of FTT to manipulate metabolism in systemic tissues prior to detectable colonization. In some cases, such as in the liver, i.n. infection caused a greater but transient response compared to the same time point assessed after i.d. inoculation. This suggested that infection route informs the magnitude of change in these systemic tissues. We cannot rule out that FTT manipulation of systemic tissues was due to contact with low numbers of circulating pathogen below the detection limit of our assays. However, a more likely explanation is that host factors responding to infection, or possibly secreted FTT virulence factors, are responsible for the metabolic effect observed. The identification and contribution of host and/or pathogen components and their impacts on systemic metabolic reprogramming is an area of ongoing exploration in our laboratory. Regardless, these data highlight that FTT and or host responses early during infection at the primary infection site can exert profound effects on metabolism in systemic tissues.

Comprehensively, this study provides a spatiotemporal atlas for both inflammatory and host metabolic responses to FTT infection in involved organ systems with respect to different infection routes. It also identified metabolic dysregulation as a key factor in FTT disease pathogenesis irrespective of where infection is first established and provided evidence that subtle differences in the early metabolic response in the lung versus dermis were strongly associated with increased virulence. Notwithstanding these findings, there remain limitations in this study that do not fully address important aspects of exposure route, metabolism, and their respective influence on disease progression. Specifically, differentiating between metabolism of the host versus FTT, especially at later stages of disease when bacterial loads reach high burdens and may significantly contribute to the total metabolite pools, remains undetermined. A systematic examination of numerous FTT mutants deficient in various metabolic pathways may help address this issue. Additionally, it is feasible that the metabolic profile of the C57Bl/6 model specifically contributes to the high sensitivity to FTT via both routes, and the use or comparison of C57Bl/6 mice to alternative strains may further assist in understanding the metabolic contributions to disease pathogenesis. Consistent with this notion, different inbred and outbred strains are reported to exhibit different levels of susceptibility to FTT infection and are reported to have different baseline metabolic profiles [45–47]. It is plausible that attenuation in these alternative strains is associated with earlier activation of metabolic reprograms at the infection site, or different metabolic demands as disease progresses. Therefore, a formal comparison between strains may further identify centrally involved metabolic functions. Secondly, due to the complexity of metabolic responses at the local and systemic level, the specific approach we used here only examined metabolite levels in isolated tissues over time despite our understanding that metabolic crosstalk between tissues is likely occurring that contributes to disease progression. The use of heavy isotope flux tracing could begin addressing organ to organ metabolic networks but requires a high degree of method development and optimization [48]. Our limited understanding of the metabolic response following infection stands as a critical barrier towards development of translational therapeutic strategies against infectious diseases. This study begins addressing this barrier and informs future design of therapeutic strategies that target tissue specific host metabolic programs manipulated by highly virulent pathogens with the objective of limiting severe disease.

## Supporting information

S1 Fig Comparative analysis of inflammatory cytokine/chemokine response following intranasal or intradermal infection. Whole tissue homogenates were evaluated for changes in cytokine/chemokine levels at 72 and 96 hours post infection (A) lungs, (B) mLN or cLN, (C) liver, (D) spleen, and (E) ear (i.d. infection only). Data shown are mean ± SEM and were pooled from two separate experiments (N = 10 mice per group). *p<0.05, **p<0.01, ***p<0.001 indicates significance between i.n. and i.d. infection using an unpaired t-test corrected for multiple comparisons using the Holm-Sidak method. For the ear after i.d. infection (B), *p<0.05, **p<0.01, ***p<0.001 indicates significance between i.d. or i.n. by students t-test corrected for multiple comparisons.
(TIF)

S2 Fig Comparative analysis of inflammatory cytokines/chemokine response following intranasal infection in the ear and cLN. Whole ear (A) or cLN (B) homogenates were evaluated for changes in cytokine/chemokine levels at over time after i.n. infection in the ear. Data shown are mean ± SEM and were pooled from two separate experiments (N = 8 mice per group). *p<0.05, ***p<0.001 indicates significance relative to mock infected control by one-way ANOVA corrected for multiple comparisons using the Dunnett method.
(TIF)

S3 Fig Annexin V-750 uptake and distribution in vivo after i.n. or i.d. FTT infection. Mice that had been infected i.d. or i.n. with FTT were injected i.v. with Annexin Vivo 750 and distribution quantified at the whole animal level (A). Tissues were removed and imaged ex vivo for tissue specific resolution of the lungs (B), ear (after i.d. infection only) (C), mLN or cLN (D), liver (E), and spleen (F). Data shown are mean +/- SEM of the average radiance efficiency and an accompanying representative image from two separate experiment (N = 10 mice per group). *p<0.05, **p<0.01, ***p<0.001 indicate significance between i.d. and i.n. infection groups using an unpaired t-test corrected for multiple comparisons using the Holm-Sidak method. #p<0.05, ##p<0.01, ###p<0.001 indicates significance within each infection group by one or 2-way ANOVA followed by Dunnett’s correction for multiple comparisons relative to mock infected controls.
(TIF)

S4 Fig Comparative analysis of ratios between raw peak LC-MS signal of NADPH/NADP+ (A-E) or NADH/NAD+ (F-J) between various tissues following i.n. or i.d. infection over time. Redox ratios comparing the lung and ear (A, F), Lung alone (B, G), mLN versus cLN (C, H), Liver (D, I), and spleen (D, I) were evaluated. Data shown are mean +/- SEM (N = 5 mice per group). *p<0.05, **p<0.01, ***p<0.001 indicate significance between i.d. and i.n. infection groups using an unpaired t-test corrected for multiple comparisons using the Holm-Sidak method. #p<0.05, ##p<0.01, ###p<0.001 indicates significance within each infection group by one or 2-way ANOVA followed by Dunnett’s correction for multiple comparisons relative to mock infected controls.
(TIF)

S5 Fig Comparative analysis of ratios between raw peak LC-MS signal of reduced/oxidized Glutathione between various tissues following i.n. or i.d. infection over time. Redox ratios comparing the lung and ear (A, F), Lung alone (B, G), mLN versus cLN (C, H), Liver (D, I), and spleen (D, I) were evaluated. Data shown are mean +/- SEM (N = 5 mice per group). *p<0.05, **p<0.01, ***p<0.001 indicate significance between i.d. and i.n. infection groups using an unpaired t-test corrected for multiple comparisons using the Holm-Sidak method. #p<0.05, ##p<0.01, ###p<0.001 indicates significance within each infection group by one or 2-way ANOVA followed by Dunnett’s correction for multiple comparisons relative to mock infected controls.
(TIF)

S6 Fig Comparative analysis of ratios between raw peak LC-MS signal of ADP/ATP and AMP/ATP ratios between various tissues following i.n. or i.d. infection over time. Redox ratios comparing the lung and ear (A, F), Lung alone (B, G), mLN versus cLN (C, H), Liver (D, I), and spleen (D, I) were evaluated. Data shown are mean +/- SEM (N = 5 mice per group). *p<0.05, **p<0.01, ***p<0.001 indicate significance between i.d. and i.n. infection groups using an unpaired t-test corrected for multiple comparisons using the Holm-Sidak method. #p<0.05, ##p<0.01, ###p<0.001 indicates significance within each infection group by one or 2-way ANOVA followed by Dunnett’s correction for multiple comparisons relative to mock infected controls.
(TIF)

S7 Fig RJ2DG uptake in the lungs (A) or ear (B) 24 hours after i.p. exposure to LPS. Data shown are representative images and accompanying graph showing average radiance efficiency. Data is shown as mean +/- SEM from data pooled from two separate experiments (N = 6 mice). *p<0.05, indicates significance between i.d. and i.n. groups using an unpaired t-test. (C) NADH/NAD+ ratio ear as detected by LC-MS. For (C) data shown is the mean +/- SEM from data pooled from two separate experiments (N = 7–9 mice).
(TIF)
