# Supplementary figures and images for: Route of Francisella tularensis infection informs spatiotemporal metabolic reprogramming and inflammation in mice
Source: PLoS One. 2023 Oct 26;18(10):e0293450. doi: 10.1371/journal.pone.0293450 (PMC10602361; doi:10.1371/journal.pone.0293450)

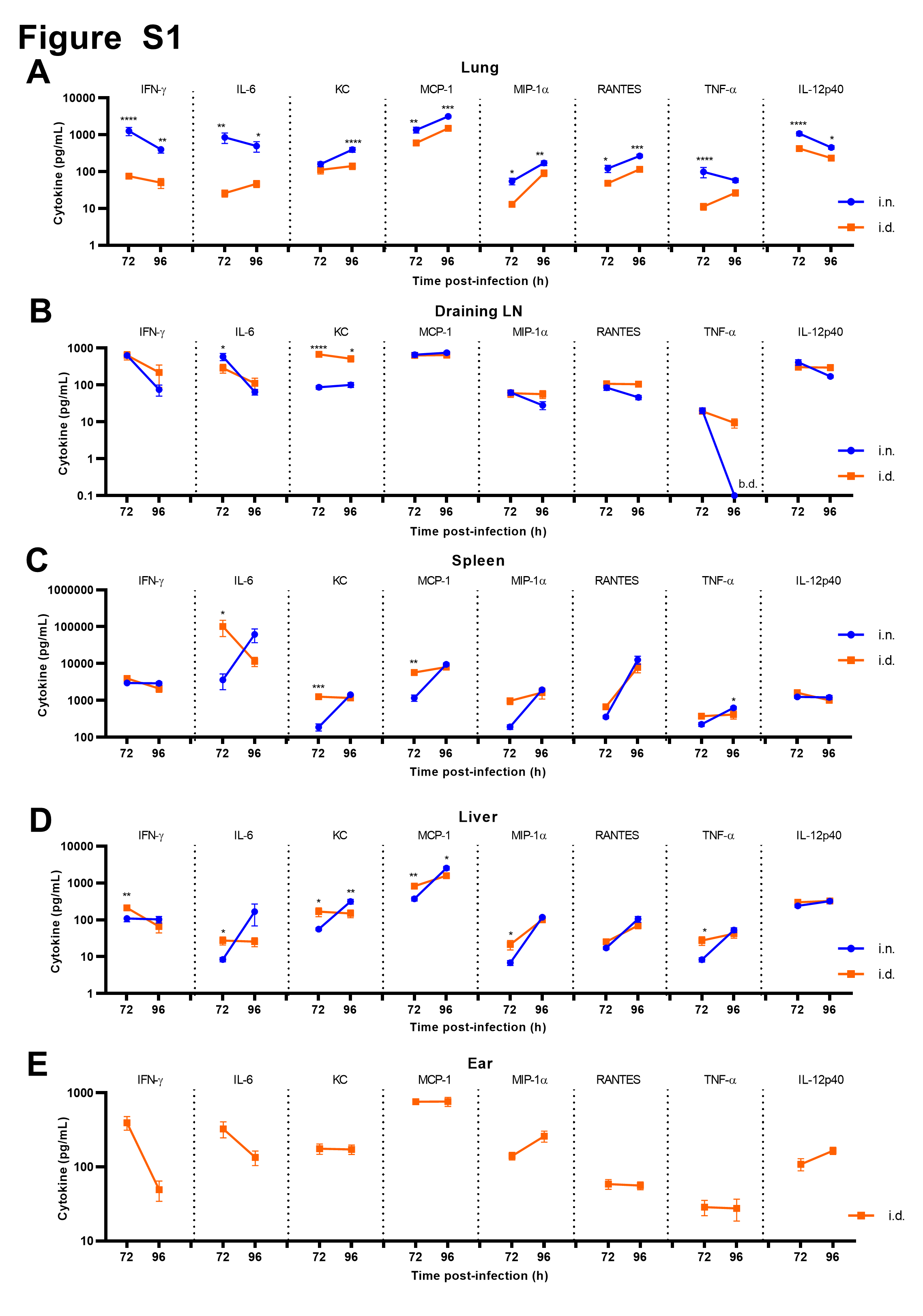

Supplement: S1 Fig — Whole tissue homogenates were evaluated for changes in cytokine/chemokine levels at 72 and 96 hours post infection (A) lungs, (B) mLN or cLN, (C) liver, (D) spleen, and (E) ear (i.d. infection only). Data shown are mean ± SEM and were pooled from two separate experiments (N = 10 mice per group). *p<0.05, **p<0.01, ***p<0.001 indicates significance between i.n. and i.d. infection using an unpaired t-test corrected for multiple comparisons using the Holm-Sidak method. For the ear after i.d. infection (B), *p<0.05, **p<0.01, ***p<0.001 indicates significance between i.d. or i.n. by students t-test corrected for multiple comparisons. (TIF) [file pone.0293450.s001.tif]

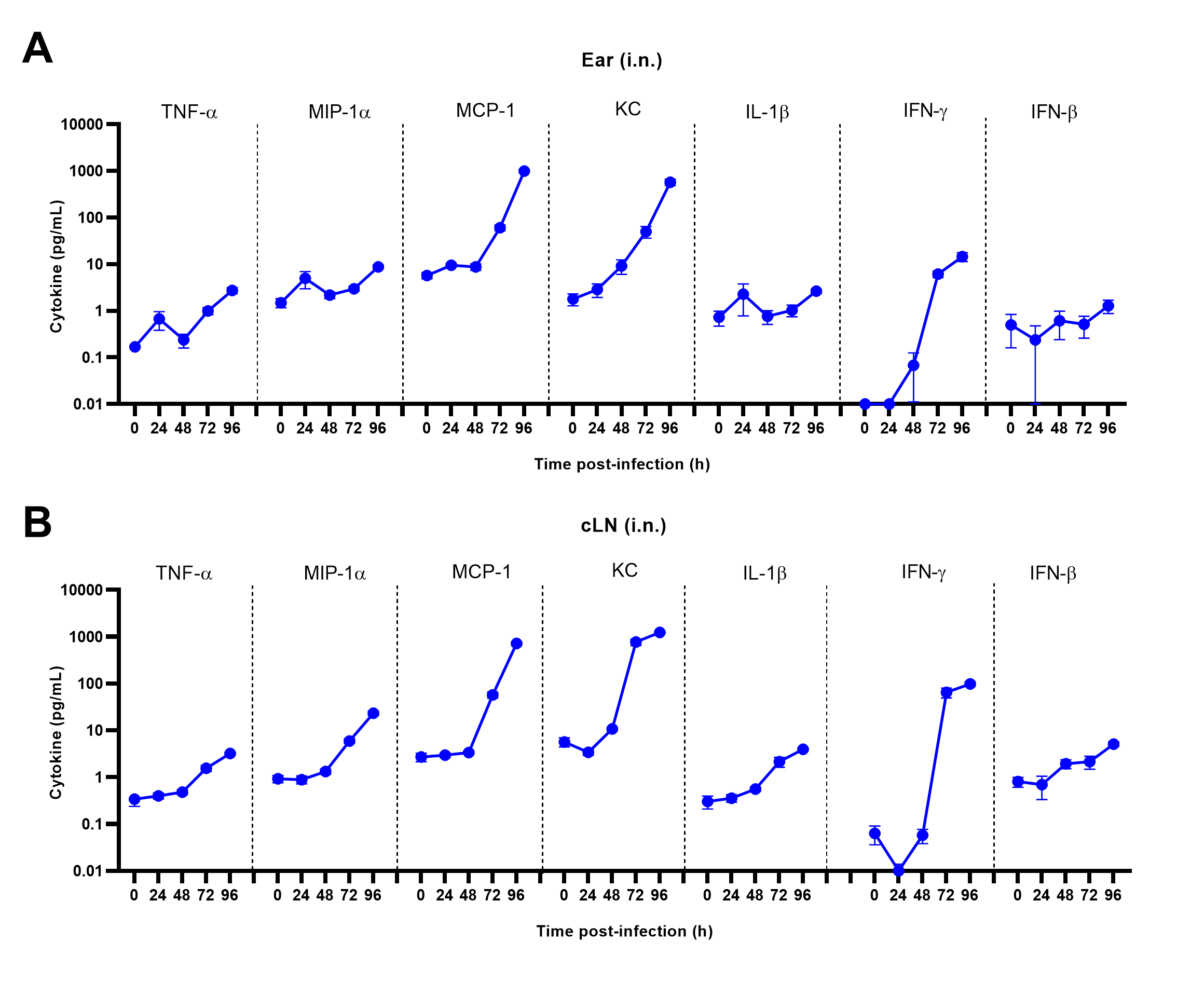

Supplement: S2 Fig — Whole ear (A) or cLN (B) homogenates were evaluated for changes in cytokine/chemokine levels at over time after i.n. infection in the ear. Data shown are mean ± SEM and were pooled from two separate experiments (N = 8 mice per group). *p<0.05, ***p<0.001 indicates significance relative to mock infected control by one-way ANOVA corrected for multiple comparisons using the Dunnett method. (TIF) [file pone.0293450.s002.tif]

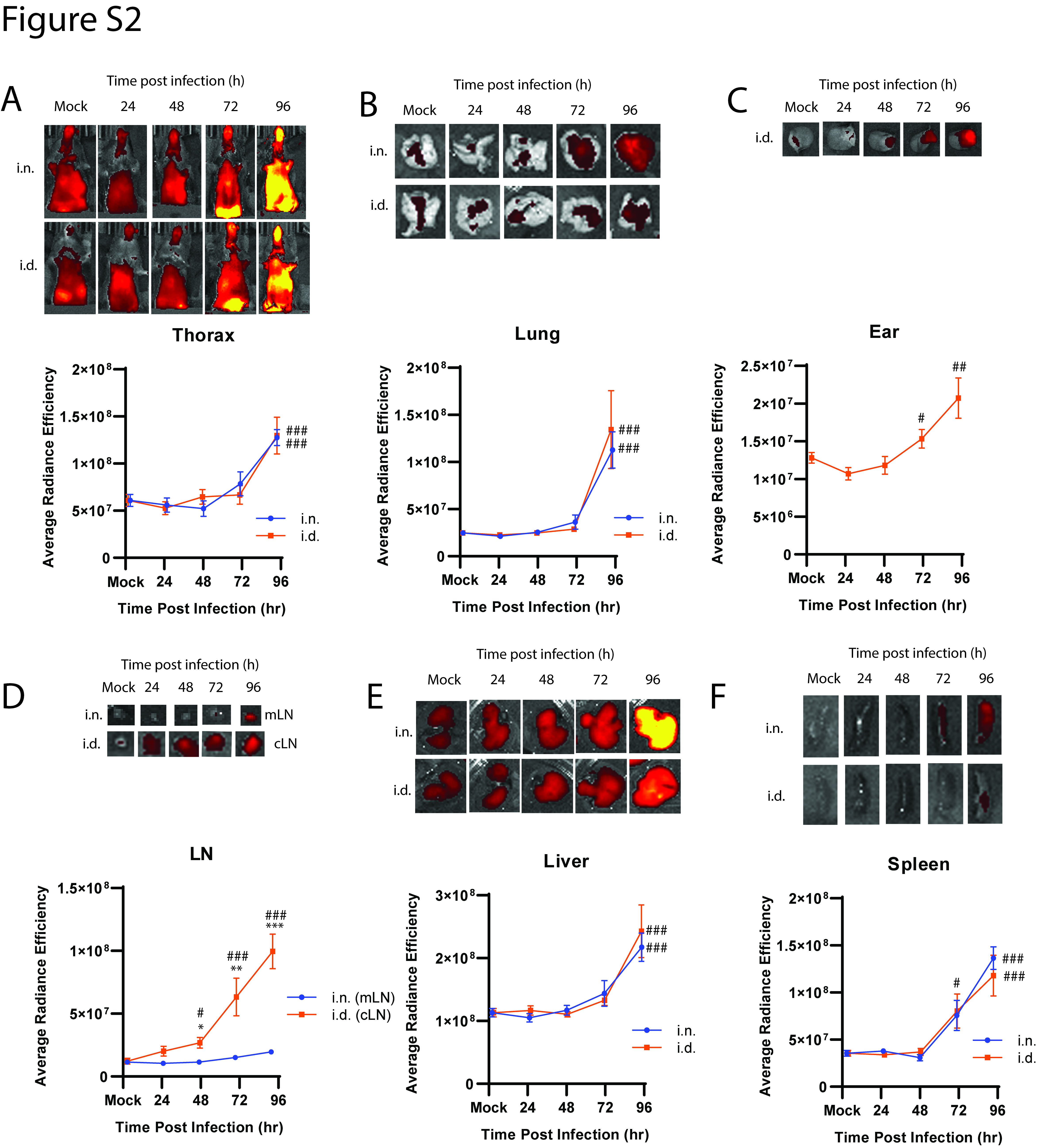

Supplement: S3 Fig — Mice that had been infected i.d. or i.n. with FTT were injected i.v. with Annexin Vivo 750 and distribution quantified at the whole animal level (A). Tissues were removed and imaged ex vivo for tissue specific resolution of the lungs (B), ear (after i.d. infection only) (C), mLN or cLN (D), liver (E), and spleen (F). Data shown are mean +/- SEM of the average radiance efficiency and an accompanying representative image from two separate experiment (N = 10 mice per group). *p<0.05, **p<0.01, ***p<0.001 indicate significance between i.d. and i.n. infection groups using an unpaired t-test corrected for multiple comparisons using the Holm-Sidak method. #p<0.05, ##p<0.01, ###p<0.001 indicates significance within each infection group by one or 2-way ANOVA followed by Dunnett’s correction for multiple comparisons relative to mock infected controls. (TIF) [file pone.0293450.s003.tif]

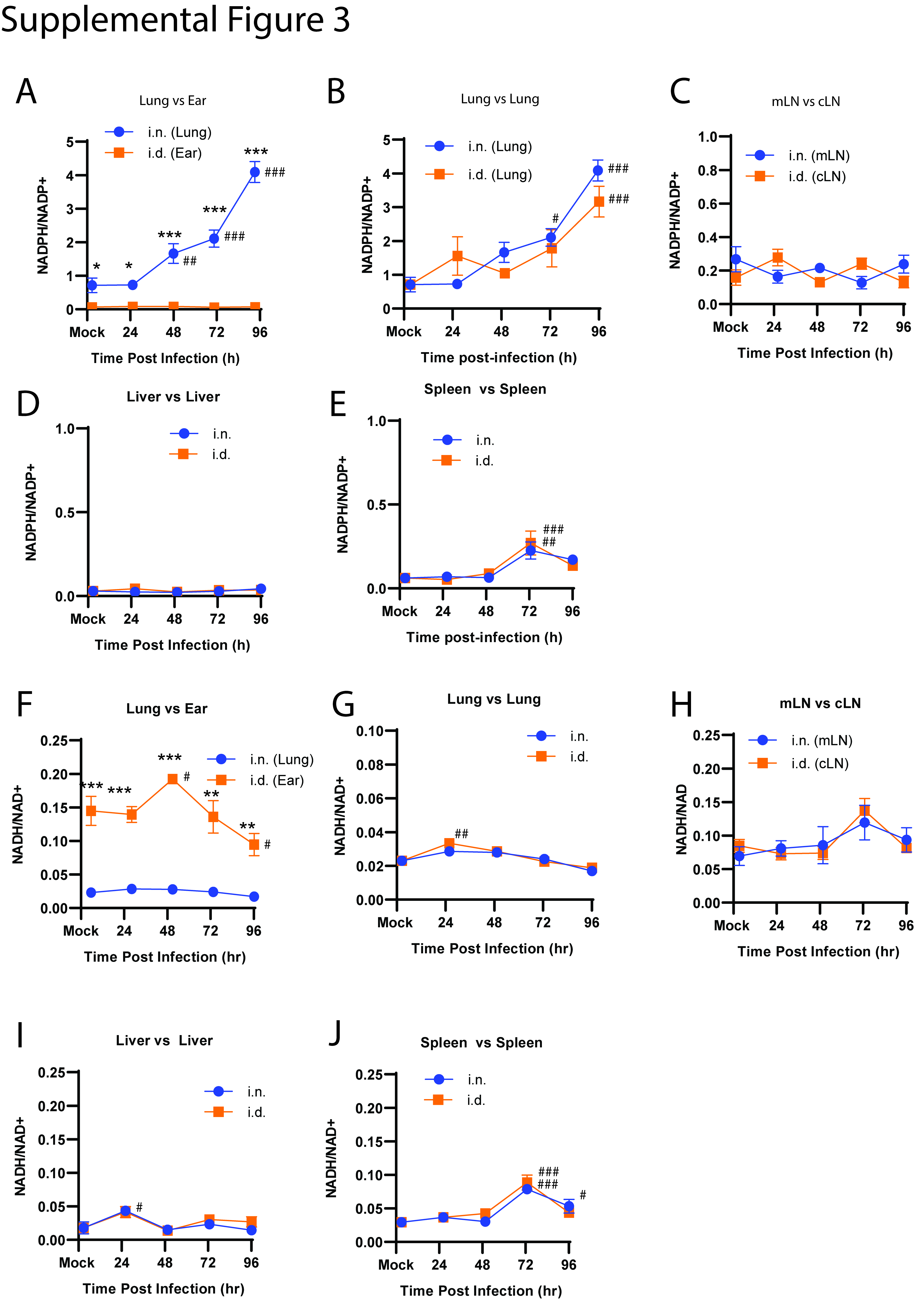

Supplement: S4 Fig — Comparative analysis of ratios between raw peak LC-MS signal of NADPH/NADP+ (A-E) or NADH/NAD+ (F-J) between various tissues following i.n. or i.d. infection over time. Redox ratios comparing the lung and ear (A, F), Lung alone (B, G), mLN versus cLN (C, H), Liver (D, I), and spleen (D, I) were evaluated. Data shown are mean +/- SEM (N = 5 mice per group). *p<0.05, **p<0.01, ***p<0.001 indicate significance between i.d. and i.n. infection groups using an unpaired t-test corrected for multiple comparisons using the Holm-Sidak method. #p<0.05, ##p<0.01, ###p<0.001 indicates significance within each infection group by one or 2-way ANOVA followed by Dunnett’s correction for multiple comparisons relative to mock infected controls. (TIF) [file pone.0293450.s004.tif]

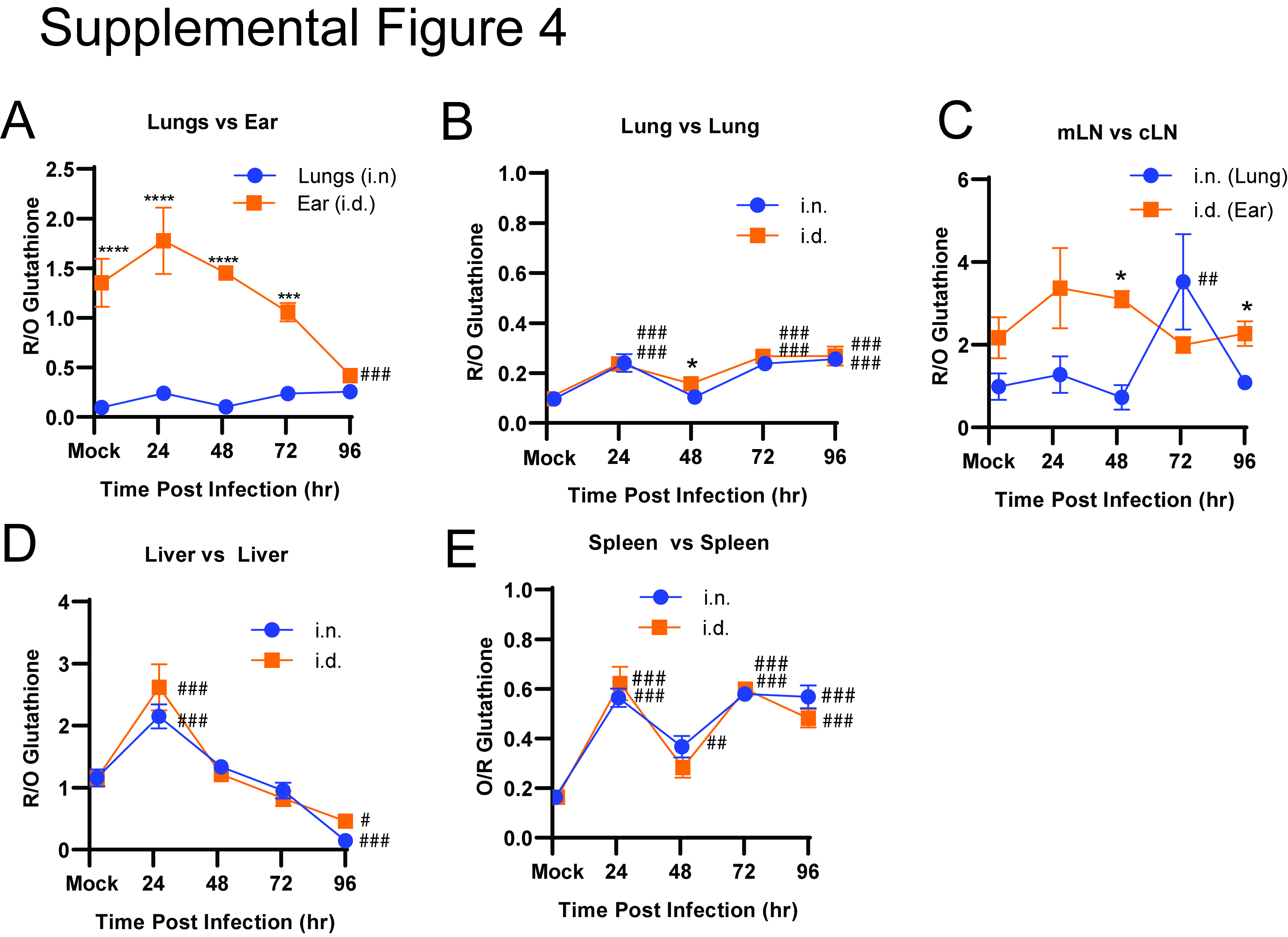

Supplement: S5 Fig — Redox ratios comparing the lung and ear (A, F), Lung alone (B, G), mLN versus cLN (C, H), Liver (D, I), and spleen (D, I) were evaluated. Data shown are mean +/- SEM (N = 5 mice per group). *p<0.05, **p<0.01, ***p<0.001 indicate significance between i.d. and i.n. infection groups using an unpaired t-test corrected for multiple comparisons using the Holm-Sidak method. #p<0.05, ##p<0.01, ###p<0.001 indicates significance within each infection group by one or 2-way ANOVA followed by Dunnett’s correction for multiple comparisons relative to mock infected controls. (TIF) [file pone.0293450.s005.tif]

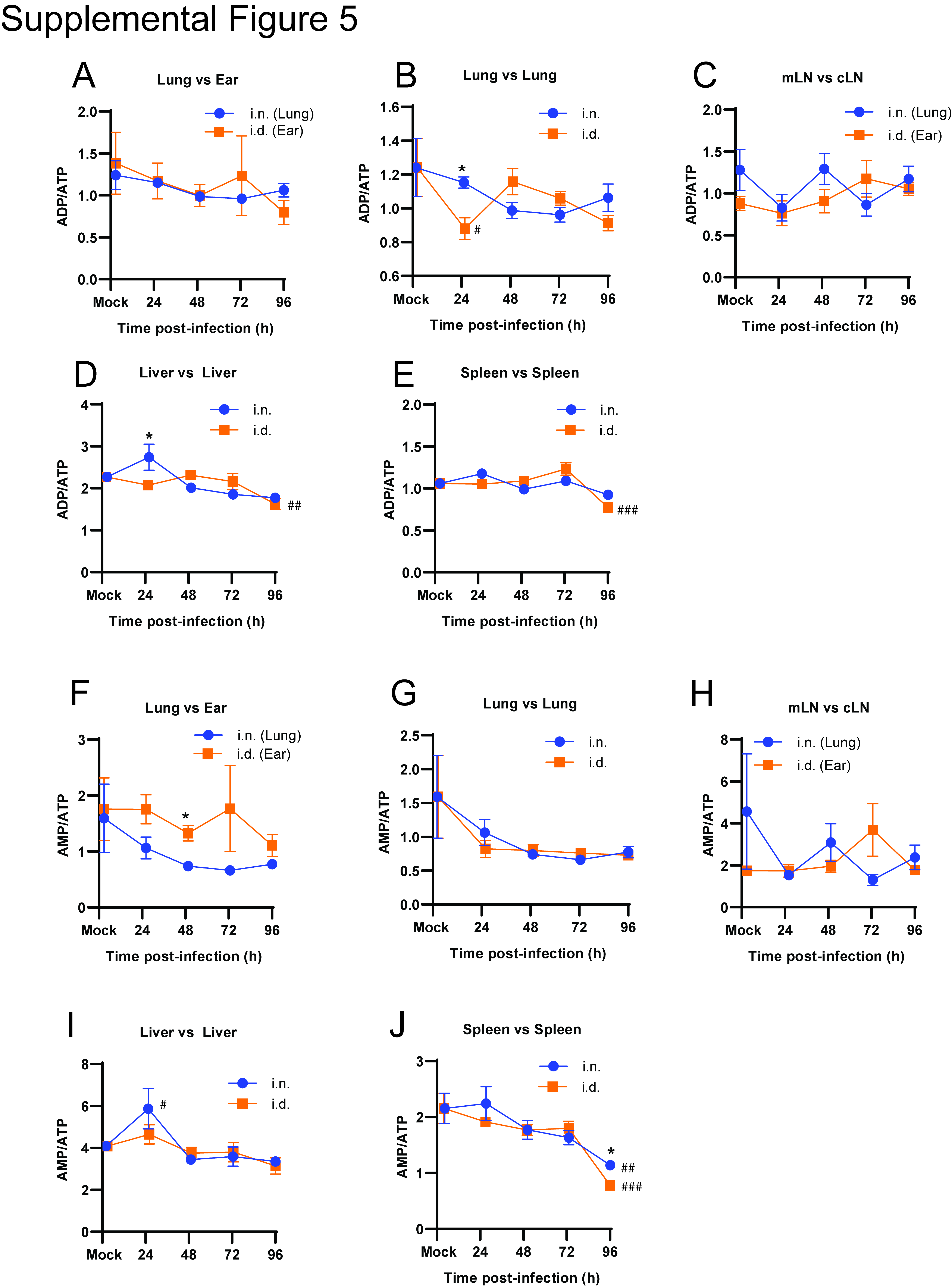

Supplement: S6 Fig — Redox ratios comparing the lung and ear (A, F), Lung alone (B, G), mLN versus cLN (C, H), Liver (D, I), and spleen (D, I) were evaluated. Data shown are mean +/- SEM (N = 5 mice per group). *p<0.05, **p<0.01, ***p<0.001 indicate significance between i.d. and i.n. infection groups using an unpaired t-test corrected for multiple comparisons using the Holm-Sidak method. #p<0.05, ##p<0.01, ###p<0.001 indicates significance within each infection group by one or 2-way ANOVA followed by Dunnett’s correction for multiple comparisons relative to mock infected controls. (TIF) [file pone.0293450.s006.tif]

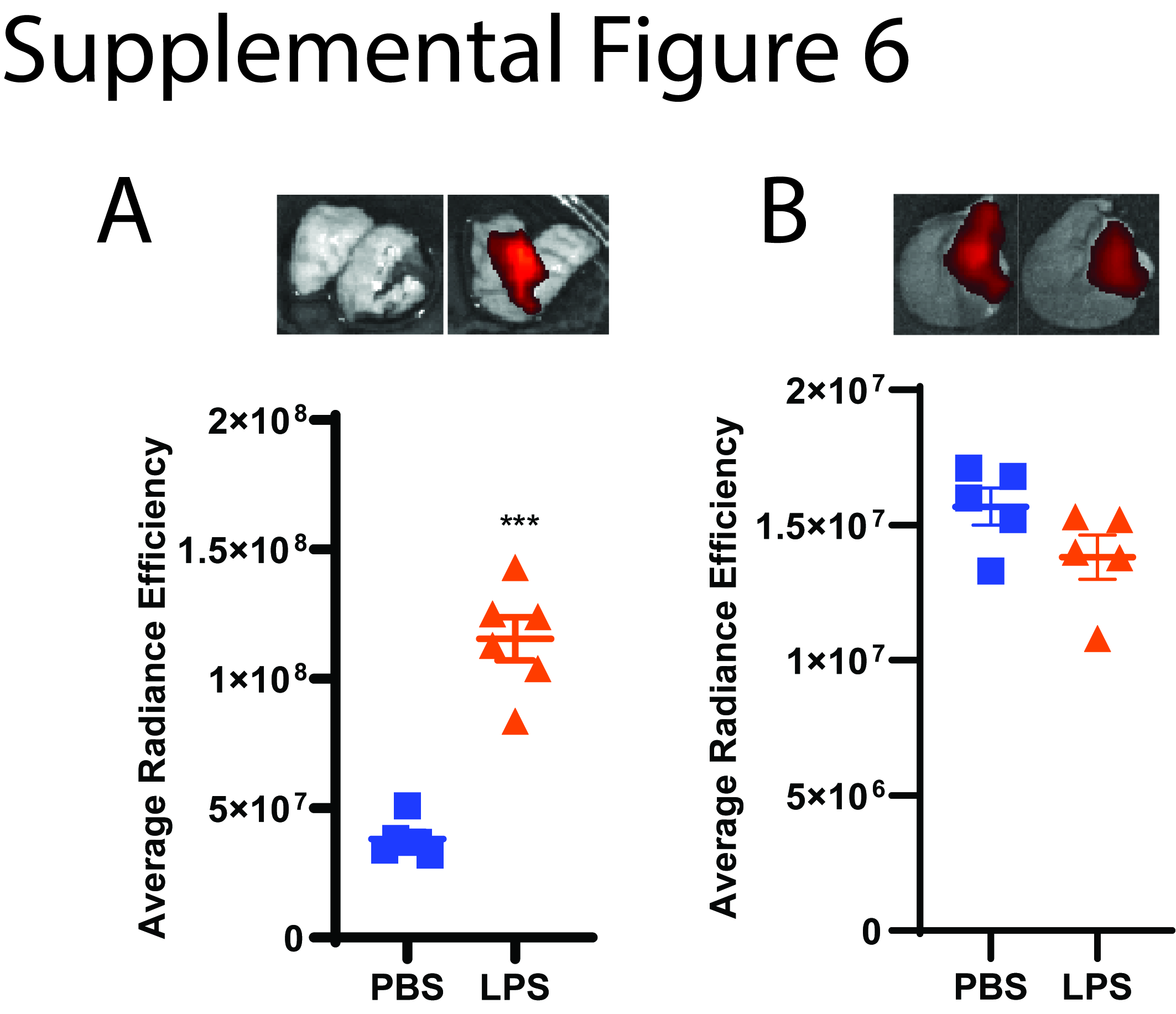

Supplement: S7 Fig — RJ2DG uptake in the lungs (A) or ear (B) 24 hours after i.p. exposure to LPS. Data shown are representative images and accompanying graph showing average radiance efficiency. Data is shown as mean +/- SEM from data pooled from two separate experiments (N = 6 mice). *p<0.05, indicates significance between i.d. and i.n. groups using an unpaired t-test. (C) NADH/NAD+ ratio ear as detected by LC-MS. For (C) data shown is the mean +/- SEM from data pooled from two separate experiments (N = 7–9 mice). (TIF) [file pone.0293450.s007.tif]
